# 3-OST-7 Regulates BMP-Dependent Cardiac Contraction

**DOI:** 10.1371/journal.pbio.1001727

**Published:** 2013-12-03

**Authors:** Shiela C. Samson, Tania Ferrer, Chuanchau J. Jou, Frank B. Sachse, Sunita S. Shankaran, Robin M. Shaw, Neil C. Chi, Martin Tristani-Firouzi, H. Joseph Yost

**Affiliations:** 1 Department of Neurobiology & Anatomy, University of Utah Molecular Medicine Program, Salt Lake City, Utah, United States of America; 2 Cardiovascular Research and Training Institute, University of Utah, Salt Lake City, Utah, United States of America; 3 Department of Bioengineering, University of Utah, Salt Lake City, Utah, United States of America; 4 Department of Medicine, Cedars-Sinai Heart Institute, University of California, Los Angeles, California, United States of America; 5 Department of Medicine, Division of Cardiology, University of California, San Diego, California, United States of America; King's College London, United Kingdom

## Abstract

The 3-O-sulfotransferase (3-OST) family catalyzes rare modifications of glycosaminoglycan chains on heparan sulfate proteoglycans, yet their biological functions are largely unknown. Knockdown of 3-OST-7 in zebrafish uncouples cardiac ventricular contraction from normal calcium cycling and electrophysiology by reducing *tropomyosin4* (*tpm4*) expression. Normal 3-OST-7 activity prevents the expansion of BMP signaling into ventricular myocytes, and ectopic activation of BMP mimics the ventricular noncontraction phenotype seen in 3-OST-7 depleted embryos. In 3-OST-7 morphants, ventricular contraction can be rescued by overexpression of tropomyosin *tpm4* but not by troponin *tnnt2*, indicating that *tpm4* serves as a lynchpin for ventricular sarcomere organization downstream of 3-OST-7. Contraction can be rescued by expression of 3-OST-7 in endocardium, or by genetic loss of *bmp4*. Strikingly, BMP misregulation seen in 3-OST-7 morphants also occurs in multiple cardiac noncontraction models, including potassium voltage-gated channel gene, *kcnh2*, affected in Romano-Ward syndrome and long-QT syndrome, and cardiac troponin T gene, *tnnt2*, affected in human cardiomyopathies. Together these results reveal 3-OST-7 as a key component of a novel pathway that constrains BMP signaling from ventricular myocytes, coordinates sarcomere assembly, and promotes cardiac contractile function.

## Abstract 2

During zebrafish cardiac development, 3-OST-7 constrains BMP signaling to the atrioventricular junction and precludes it from contractile myocardium, allowing tropomyosin-dependent sarcomere assembly and contraction.

## Author Summary

A highly complex environment at the cell surface and in the space between cells is thought to modulate cell behavior. Heparan sulfate proteoglycans are cell surface and extracellular matrix molecules that are covalently linked to long chains of repeating sugar units called glycosaminoglycan chains. These chains can be subjected to rare modifications and they are believed to influence specific cell signaling events in a lineage specific fashion in what is called the “glycocode.” Here we explore the functions of one member of a family of enzymes, 3-O-sulfotransferases (3-OSTs) that catalyzes a rare modification (3-O-sulfation) of glycosaminoglycans in zebrafish. We show that knockdown of 3-OST-7 results in a very specific phenotype, including loss of cardiac ventricle contraction. Knockdown of other 3-OST family members did not result in the same phenotype, suggesting that distinct 3-OST family members have distinct functions in vertebrates and lending *in vivo* evidence for the glycocode hypothesis. Mechanistically, we found that cardiac contraction can be rescued by reducing the amount of endogenous BMP4, and can be blocked by increasing BMP signaling, suggesting that the glycocode generated by 3-OST-7 is necessary to constrain BMP signaling in the heart for normal cardiac contraction. Furthermore, we show that *tropomyosin4* (tpm4) is downstream of 3-OST-7 function, indicating that Tpm4 is key in this pathway to building the sarcomere, the functional contraction unit of the cardiomyocyte.

## Introduction

Vertebrate heart development requires an accurate integration of patterning and morphogenetic events, leading eventually to the formation of a fully functional heart. It initiates with the specification of the different tissue lineages that will compose the mature heart, followed by an intricate set of differentiation events that will transform the early heart field into a mature, beating organ. This transformation is defined by the subspecialization of regions of the primitive heart tube to acquire characteristics of contractile myocardium or region-specific maintenance of noncontracting myocardium. These complex events are orchestrated by a network of signals and transcription factors that could act differentially depending upon specific spatiotemporal cues. Among the important players are major signaling pathways such as BMP signaling and Wnt signaling, which set the early stages of differentiation [1]–[4], and the T-box (Tbx) family of transcription factors that confer chamber or nonchamber identity to the primitive heart tube [5].

Ultimately, generation of a beating heart is the goal of these processes. For the heart to contract, contractile proteins must be produced and assembled into sarcomeres and their contraction must be coupled to the cycling electrophysiological activity. The heart starts beating during the late stages of heart tube formation and through its mechanical action, affects subsequent differentiation steps as shown in studies correlating defective morphogenesis with abnormal function [6]–[9]. Despite this understanding of heart development, critical questions remain in the field and unknown players remain to be discovered. In this study, we focus on the role in heart development of an enzyme, 3-O-sulfotransferase-7 (3-OST-7), that modifies heparan sulfate proteoglycans (HSPGs).

HSPGs are cell surface and extracellular matrix (ECM) molecules composed of a core protein to which glycosaminoglycan (GAG) chains are covalently linked. The ability of HSPGs to interact with signaling ligands and receptors and ECM components place them at a unique advantage to modulate complex biological processes such as morphogenesis, tissue repair, and host defense [10]–[12]. The specificity of interactions of an HSPG and its environment is due, in part, to the GAG chains [12]–[14]. The GAG chains in HSPGs are unbranched, charged polysaccharides composed of 50 or more repeating disaccharide units of *N*-acetylglucosamine and glucuronic acid. These chains are subjected to several kinds of modifications: *N*-deacetylation/*N*-sulfation, epimerization, and O-sulfation. Not all disaccharide residues are modified, resulting in GAG chains with relatively small clusters of modified units interspersed among large sections of unmodified units [15]. This gives rise to an astounding level of structural heterogeneity, producing GAG chains with varying specificities for protein binding [10]. The repertoire of modifying enzymes differs between cells [10], which in theory could impact how a cell interacts with a ligand, a neighboring cell, or the ECM. Previous gene knockdown and knockout studies have begun to document the roles for these modifying enzymes [14], but none has been implicated in heart development.

In this study, we focus on a rare and specific kind of O-sulfation, 3-O-sulfation, performed by a family of enzymes, the 3-O-sulfotransferases (3-OSTs). 3-OSTs catalyze transfer of a sulfate group to the hydroxyl of the third carbon of *N*-sulfated glucosamine residues [15]. Previous work in our lab has identified and cloned the 3-OST family in zebrafish [16]. Gene expression analysis reveals dynamic spatial and temporal expression patterns for the eight 3-OST family members suggesting distinct roles in the developing embryo.

Here we show that morpholino (MO) knockdown of one of eight 3-OST family members in zebrafish, 3-OST-7 (aka *hs3st1l1*), specifically results in a noncontracting cardiac ventricle at 48 hours post fertilization (hpf). Surprisingly, electrical and calcium transients in cardiomyocytes appear to be normal, suggesting that normal electrophysiological signaling in cardiomyocytes is uncoupled from cardiomyocyte contraction. Further exploring the noncontracting phenotype, we show that 3-OST-7 functions to negatively regulate BMP signaling in cardiomyocytes and to allow *tpm4* mRNA accumulation, which then allows normal sarcomere organization and contraction. The roles of 3-OST-7 and BMP signaling reveal a novel mechanism for the regulation of cardiac cell function.

## Results

### Cardiac Ventricle Contraction Requires 3-OST-7

**Figure 1:**
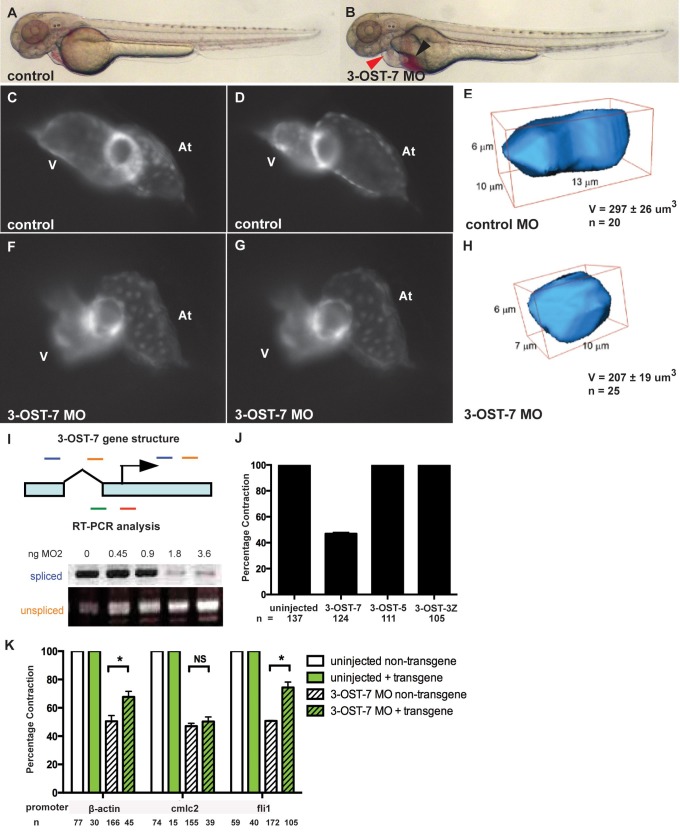
3-OST-7 is required for ventricular contraction in the zebrafish heart. Lateral views of control (uninjected, wild-type) (A) and 3-OST-7 morphant (B) embryos at 48 hpf showing edema (red arrowhead) and blood pooling (black arrowhead) with knockdown of 3-OST-7. Lateral views of hearts in control (C–D) and 3-OST-7 morphant (F–G) *Tg(cmlc2:gfp)* embryos at periods of ventricular diastole (C and F) and systole (D and G). 3D-reconstructed ventricular cell in control (E) and 3-OST-7 morphant (H) embryos show decreased volume (V) in morphants. (I) Gene structure of 3-OST-7 showing MO targets (red for MO1 and green for MO2) and primer sets used for RT-PCR analysis (blue and orange bars). RT-PCR analysis showing decrease in spliced and increase in unspliced products with increasing dose of MO injected. (J) Percentage contraction of embryos in different 3-OST knockdowns (3-OST-7, 3-OST-5, 3-OST-3Z). Only knockdown of 3-OST-7 MO resulted in ventricular noncontraction. (K) Percentage contraction of embryos in rescue experiments using three different transgenes. Uninjected embryos with (green bar) or without (white bar) the transgene had normal ventricular contraction. Injection of 3-OST-7 MO2 in *Tg(β-actin:3-OST-7-IEP)* and *Tg(fli1:3-OST-7-IEP)* embryos rescued the noncontracting ventricle phenotype (*p* = 0.039 and *p* = 0.0036, respectively, *). Injection of 3-OST-7 MO2 in *Tg(cmlc2:3-OST-7-IEP)* did not rescue the phenotype (*p* = 0.44, NS). Graphs depict the percentage of embryos with phenotype in 3-OST-7-overexpressing (GFP+, green hatched) or non-3-OST-7-overexpressing (GFP-, white hatched) embryos from individual crosses between one of three transgenic founders and wild-type AB zebrafish. At, atrium; V, ventricle; error bars, SEM.

To begin elucidating the role of 3-OST-7 in development, we injected zebrafish embryos with either a translation-blocking MO (MO1) or a splice-blocking MO (MO2). Knockdown with MO2 was verified by reverse transcription (RT)-PCR analysis (Figure 1I). Embryos injected with either MO exhibited similar phenotypes indicative of a cardiovascular phenotype: pericardial edema and blood pooling at 48 hpf (Figure 1A and 1B). Visualizing the heart in living transgenic *Tg(cmlc2:gfp)* zebrafish [17] revealed that 3-OST-7 morphants had a hypoplastic cardiac ventricle that did not contract normally (Figure 1F and 1G; Video S2), resulting in poor blood circulation (Video S4). In contrast, sibling control embryos had normal cardiac contraction cycles, with sequential diastole and systole, and normal blood circulation (Figure 1C and 1D, Videos S1 and S3). In contrast to the ventricle in 3-OST-7 morphants, atrium contraction was normal and similar to control (Figure 1C, 1D, 1F, and 1G). In embryos injected with MO2, only 47%±1% (*n* = 124) had normal ventricular contraction, whereas all control embryos had normal cardiac contraction (*n* = 137) (Figure 1J).

To assess the specificity of 3-OST-7 knockdowns, we injected MOs against two other members of the 3-OST family, 3-OST-5 and 3-OST-3z, and found that MO-injected embryos had normal cardiac ventricular contraction (Figure 1J). Knockdown of 3-OST-5 and 3-OST-6 resulted in other distinct phenotypes, including altered cilia function and left-right patterning [18]. Together, these results indicate that ventricular cardiac contraction defects are a specific phenotype of 3-OST-7 knockdown, and not knockdown of other members of the 3-OST family, including 3-OST-5, a member of the same subgroup as 3-OST-7.

In order to determine what is causing the hypoplasticity of the 3-OST-7 morphant ventricle, we examined cell number and cell volume. Utilizing the transgene *Tg(cmlc2-DsRed-nuc)* [19], which labels cardiomyocyte nuclei, we counted the total number of cardiomyocytes in both control embryos and 3-OST-7 morphants. The cardiomyocyte cell number was similar in control (298±10, *n* = 10) and 3-OST-7 morphant (288±12, *n* = 10), indicating that changes in cell number were not the cause for the hypoplastic ventricle in 3-OST-7 morphants. In contrast, by measuring ventricular myocyte volume in 3D-reconstructions of optical sections of the cardiac tube, we found that the cellular volume of individual ventricular myocytes was significantly reduced in 3-OST-7 morphants compared to control embryos (Figure 1E and 1H, *p* = 0.01), thus suggesting that cell shape changes were correlated with the observed hypoplasticity of 3-OST-7 morphant ventricles. Individual atrial myocyte volume was similar (*p* = 0.10) between control (207±11 µm^3^, *n* = 7) and 3-OST-7 morphant (183±11 µm^3^, *n* = 10).

### Expression of 3-OST-7 in Endocardium Rescues Contraction in Myocardium

When using MOs to knockdown gene function, an important control is to rescue MO phenotype by co-expression of the targeted gene. We utilized the Tol2kit cloning system [20] to create stable, germline-transmitted transgenic *Tg(β-actin:3-OST-7-IEP)* zebrafish that expressed 3-OST-7 under the control of the *β-actin* promoter for ubiquitous expression throughout early development. To preclude inhibition of transgenic expression of 3-OST-7, the MO binding sequence is not present in the construct. We placed an IRES-EGFP-polyA (IEP) downstream of the 3-OST-7 coding region, which enabled identification of individual transgenic embryos expressing 3-OST-7 by co-expression of EGFP. Constitutive expression of 3-OST-7 reduced the contraction defect in 3-OST-7 morphants, compared to nontransgenic morphants (Figure 1K).

3-O-sulfotransferases modify HSPGs, which typically function at the cell surface, making it possible that they modulate cell-cell signaling into 3-OST-expressing cell and/or into neighboring cells. 3-OST-7 is expressed ubiquitously early in development [16]. In the ventricle at 48 hpf, the myocardium is directly apposed to the endocardium, so either cell lineage could be a source for 3-OST-7 function. To refine our understanding of 3-OST-7 in cardiac ventricular contraction, we asked which ventricular cell lineage required 3-OST-7 in order to allow normal ventricular contraction. We created stable transgenic zebrafish lines that express 3-OST-7 under the control of the *cmlc2* (aka *myl7*) promoter in myocardial-specific lineages, or expressing 3-OST-7 under the control of the fli1 promoter in endothelial/endocardial-specific lineages. The 3-OST-7 MO binding sequence is not present in either construct, therefore the MO would not inhibit transgenic expression of 3-OST-7. Both transgenes have an IRES-EGFP tag downstream of 3-OST-7 coding region, enabling identification of embryos that express 3-OST-7 in either endocardium or myocardium by co-expression of EGFP. These transgenic embryos were injected with 3-OST-7 MO and compared with nontransgenic, MO-injected sibling embryos. We observed significant rescue of ventricle contraction in morphants in which 3-OST-7 was expressed in endocardium, *Tg(fli1:3-OST-7-IEP)*, that was comparable to rescue by ubiquitously expressed 3-OST-7 in transgenic *Tg(β-actin:3-OST-7-IEP)* morphants (Figure 1K). In contrast, transgenic expression of 3-OST-7 in cardiomyocytes by *Tg(cmlc2:3-OST-7-IEP)* was not sufficient to rescue cardiac contraction (Figure 1K). While the *fli1* driver rescues to the same extent as the ubiquitous driver, it is possible that other tissues might also utilize 3-OST-7. Also, it should be noted that it is not possible to conclude that 3-OST-7 is *not* also required in cardiomyocytes earlier in development. Transgenic *cmlc2* expression begins at approximately 16 hpf, while the *fli1*-driven transgene starts being expressed at approximately 12 hpf, as assessed by co-expression of EGFP with 3-OST-7 (unpublished data). Thus, it is possible that the inability of the *cmlc2:3-OST-7* transgene to rescue is due to expression that is too late to be effective. Nonetheless, these results indicate that expression of 3-OST-7 in endocardium is *sufficient* to rescue the contraction of myocardium, suggesting 3-OST-7 functions to regulate cell-cell signaling across these apposed tissues.

### Early Patterning in 3-OST-7 Morphants Is Normal

We explored several possible causes of ventricular noncontraction in 3-OST-7 morphants, including alterations in cardiac patterning or cardiomyocyte development. We used *in situ* hybridizations (ISH) and transgenic fish to assess whether the heart field is correctly specified in 3-OST-7 morphants. *Hand2* and *nkx2.5*, whose combined expressions define cardiac precursor cells in the lateral plate mesoderm [21], have similar expression patterns in both control embryos and 3-OST-7 morphants (Figure S1A–S1D). Similarly, expression patterns of *cmlc2* (myocardial marker), *amhc* (atrial marker), and *vmhc* (ventricular marker) were unaltered in 3-OST-7 morphants (Figure S1E and S1F, S1I–S1L). Endocardial precursor patterning was similar in control and 3-OST-7 embryos (Figure S1G and S1H), as assessed in *Tg(fli1:EGFP embryos)* [22]. Together these results demonstrate that heart field specification, early endocardial development, and myocardial development proceed normally in 3-OST-7 morphants, and that early mispatterning is not likely the cause for the noncontracting ventricle.

### Noncontracting Ventricle in 3-OST-7 Morphants Generates Normal Action Potentials and Calcium Transients

**Figure 2:**
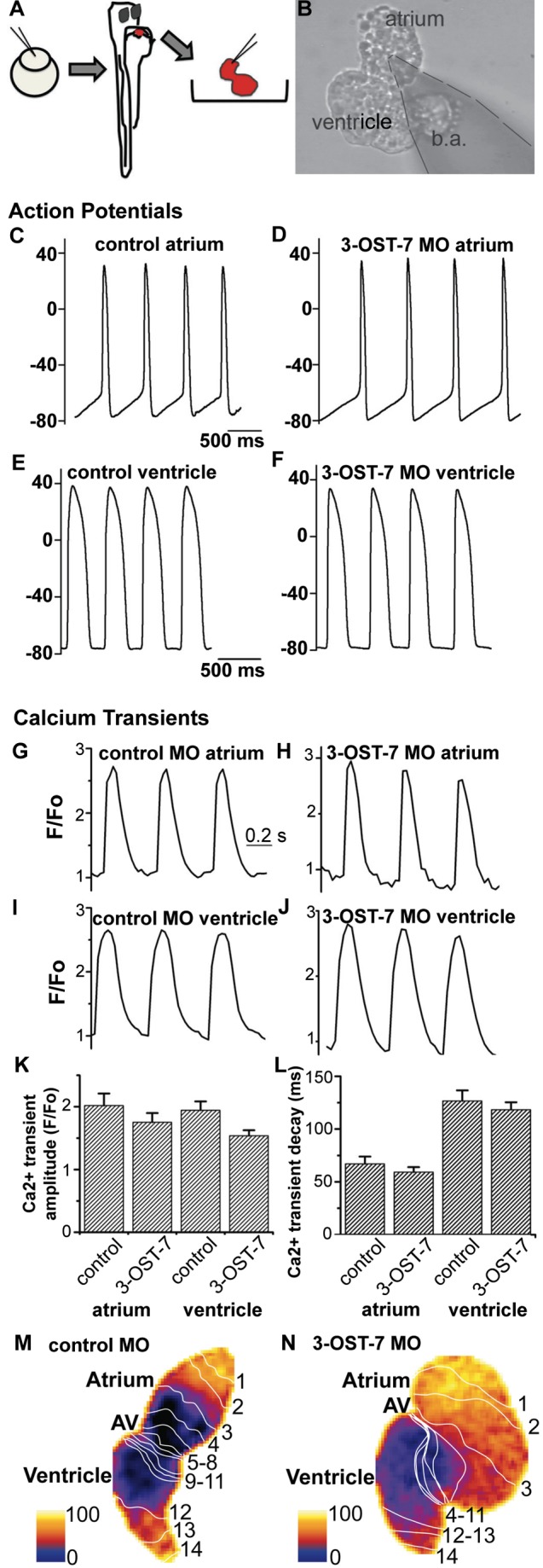
Noncontracting ventricle in 3-OST-7 morphants generates normal action potentials and calcium transients. (A) Zebrafish embryos were injected with 3-OST-7 MO1 at one-cell stage and allowed to develop until 48 hpf. The heart was then dissected out of the embryo and either placed in a recording chamber perfused with external control solution (for action potential analysis) or incubated in Fluo-4 and imaged using a confocal microscope (for calcium transient analysis). Control 48 hpf hearts from uninjected embryos or injected with control 3-OST-5 MO were processed in parallel. (B) A suction pipette was used to patch clamp the heart at the atrial or ventricular region. Action potentials were recorded from atria (C–D) and ventricles (E–F) of control (uninjected, WT) (C and E) and 3-OST-7 morphant (D and F) embryos. The action potentials were comparable between control and 3-OST-7 morphant embryos. Regions were selected from atrium and ventricle to record the calcium transients (G–J) and measure the Ca^2+^ transient amplitude (K) and Ca^2+^ transient decay (L) (error bars, SEM). No significant difference was detected between control and 3-OST-7 morphant embryos (*p* = 0.30 for atrial transient amplitude; *p* = 0.16 for ventricular transient amplitude; *p* = 0.37 for atrial transient decay; and *p* = 0.51 for ventricular transient decay). In the second technique to image the calcium transients, 3-OST-7 MO1 or control 3-OST-3Z MO was injected into *Tg(cmlc2:gCaMP)^s878^* embryos at one-cell stage and allowed to develop until 48 hpf. Optical maps of calcium activation for a single cycle, represented by isochronal lines every 20 ms, show calcium activation and conduction velocity proceed normally in 3-OST-7 morphants (N) compared to control MO injected embryos (M). Conduction proceeds from the atrium through the AV canal to the ventricle and numbers indicate the temporal sequence of calcium activation. Color bar chart fluorescence intensity changes on a scale of 0 to 100. b.a., bulbus arteriosus.

To determine whether ventricular noncontraction in 3-OST-7 morphants was due to defects in cardiomyocyte physiology, we assessed coupling of contraction to excitation. A fully functional heart characteristically undergoes excitation-contraction coupling, a physiological process whereby an electrical stimulus (action potential) is converted to a mechanical response (contraction) [23]. We first assessed whether the morphant ventricle could generate action potentials and calcium transients. To record action potentials, we performed patch clamp analysis on either the atrium or ventricle (Figure 2A and 2B) as previously described [24]. As expected, atria of 3-OST-7 morphants generated action potentials comparable to atria of control embryos (Figure 2C and 2D). Surprisingly, however, action potentials were also obtained for the noncontracting ventricles of 3-OST-7 morphants and these action potentials were similar to those recorded for ventricles of control embryos (Figure 2E and 2F). Moreover, analysis of action potential parameters revealed that there were no statistically significant differences between control embryos and 3-OST-7 morphants (Tables S1 and S2). These results indicate that the ion channels responsible for generating and propagating these action potentials were intact and physiologically functional in 3-OST-7 morphants.

A primary function of the cardiac action potential is to trigger the increase in intracellular calcium that initiates cardiac contraction [25]. To assess whether this increase occurs in 3-OST-7 morphant ventricles, we used two different techniques to image changes in intracellular calcium. In the first technique, explanted embryonic hearts were imaged by high-speed confocal microscopy using the calcium indicator Fluo-4. The amplitude and the decay of recorded calcium transients were measured to assess the release and re-uptake of intracellular calcium. Similar calcium waves were observed in hearts of both control embryos and 3-OST-7 morphants (Videos S5 and S6). The 3-OST-7 morphant atria and ventricles generated calcium transients (Figure 2H and 2J) similar to those generated by atria and ventricles from control embryos (Figure 2G and 2I). There were no significant differences in the calcium transient amplitude and calcium transient decay between ventricles of 3-OST-7 morphant embryos and ventricles of control embryos (Figure 2K and 2L). In the second technique, 3-OST-7 MO or control MO was injected into transgenic *Tg(cmlc2:gCaMP)^s878^* embryos [26] that allowed for live calcium imaging in intact zebrafish. Similar to the other technique, calcium waves were detected in 3-OST-7 morphant hearts (Videos S7 and S8) and comparable optical maps were generated for both control embryos and 3-OST-7 morphants (Figure 2M and 2N). There were no observed differences in conduction velocity. Together these results demonstrated the ability of the noncontracting ventricle of 3-OST-7 morphants to release calcium from the sarcoplasmic reticulum and to re-uptake it at the end of the cycle. These results indicate that the intracellular components that are critical for calcium cycling are functional in 3-OST-7 morphants. In addition, the normal propagation of calcium waves in 3-OST-7 morphant hearts indicates that the gap junctions and excitatory ion currents critical for normal cell-cell conduction were also intact.

### 3-OST-7-Dependent Expression of *Tpm4* Is Required for Myofibrillogenesis, Sarcomere Assembly, and Contraction

**Figure 3:**
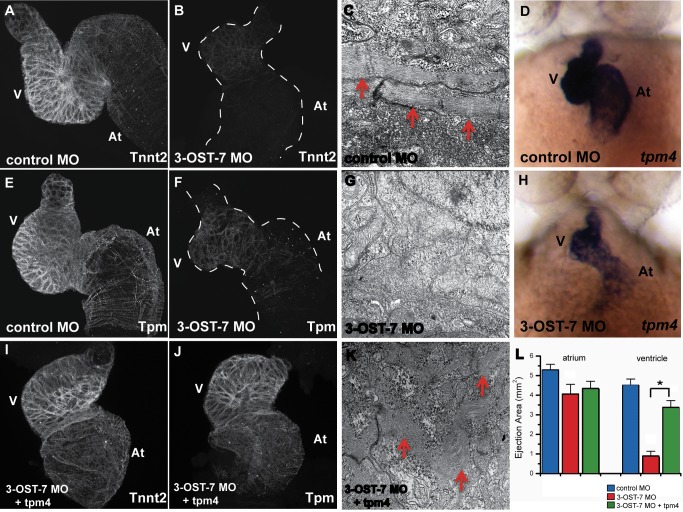
3-OST-7 regulates *tpm4*-driven myofibrillogenesis, sarcomere assembly and ventricular contraction. Whole mount IHC was performed on fixed 48(injected with control 3-OST-3Z MO) or 3-OST-7 morphant embryos to detect cardiac sarcomere proteins (*n* = 30 for each group). The heart was then dissected out of the embryo, mounted on cover slips, and imaged using a confocal microscope (thus, the dorso-ventral orientation of the mounted hearts was random). IHC using anti-Tnnt2 antibody and anti-Tpm antibody revealed levels of these proteins were greatly reduced in 3-OST-7 morphants (B and F, dashed lines outline the hearts) compared to control (A and E) embryos. TEM of control (C) and 3-OST-7 morphant (G) hearts show the presence of organized myofibrils (red arrowheads) in control and absence in morphants. ISH for *tpm4* showed *tpm4* transcript levels were decreased in 3-OST-7 morphants (H) compared to control embryos (D) at 48 hpf. (D and H) are ventral views with anterior on top; *n* = 40 for each group. Overexpression of *tpm4* rescues the expression of Tnnt2 (I) and Tpm (J) proteins, assembly of myofibrils (K), and the noncontracting ventricle phenotype in 3-OST-7 morphant embryos as assessed by ejection area measurements (L, *p*<0.05, *). The ejection area, a measure of contractility, was obtained by computing the difference between systolic and diastolic area for either atrium or ventricle. At, atrium; V, ventricle; error bars, SEM.

Our observations that action potentials and calcium transients were normal in 3-OST-7 morphants with noncontracting ventricles indicate that excitation was uncoupled from contraction. Moreover, this suggests that the failure of contraction in 3-OST-7 morphants might be due to defects in the myocardial contractile apparatus, which is the direct target of calcium ions released from the sarcoplasmic reticulum during electrical excitation of the heart. To determine whether ventricular noncontraction of 3-OST-7 morphants is due to aberrant sarcomeres, we used immunohistochemistry (IHC) and transmission electron microscopy (TEM) to visualize the sarcomeric structure of the heart. Using MF20 and phalloidin to stain sarcomeric myosin and the actin filaments, respectively, we found that these filaments were disorganized in 3-OST-7 morphant hearts compared to the orderly filament organization in hearts of control embryos (Figure S2). IHC analysis also showed diminished cardiac troponin T (Tnnt2) and tropomyosin (Tpm) organization in 3-OST-7 morphant hearts (Figure 3B and 3F) compared to control embryo hearts (Figure 3A and 3E). Myofibrils with distinct sarcomeric structures such as A-bands, I-bands, and Z-discs were evident by TEM in control hearts (Figure 3C). In contrast, the myofibrils were reduced and disorganized in 3-OST-7 morphant hearts (Figure 3G). Together these results demonstrate the noncontraction of the ventricle in 3-OST-7 morphants is correlated with disorganization of sarcomere proteins.

Since it appears that 3-OST-7 is required for sarcomere organization, and for Tnnt2 and Tpm protein levels (Figure 3B and 3F), we asked whether 3-OST-7 MO affects RNA transcript accumulation for either of these sarcomeric genes. ISH analysis of *tnnt2* RNA expression at 20 hpf, 24 hpf, and 48 hpf in morphants revealed that *tnnt2* transcript levels were similar to control (Figure S3A, S3B, S3E, S3F, S3I, S3J). In contrast, transcript levels of *tpm4* were reduced in 3-OST-7 morphants compared to control embryos (Figures 3D, 3H, S3C, S3D, S3G, and S3H). In hearts obtained by bulk disruption of 48 hpf embryos [27], *tpm4* transcript levels were reduced 3.8-fold in 3-OST-7 morphants (*p* = 6.2×10^−4^), as assessed by microarray analysis. Similar to ISH data, *tnnt2* transcript levels were unchanged in the microarray analysis (*p*>0.05). Together, these results suggest that 3-OST-7 MO leads to a reduction of *tpm4* RNA accumulation, which then leads to reduced Tpm protein accumulation.

We suggest that cardiac Tpm4 serves as a “lynchpin” protein downstream of 3-OST-7 function; when Tpm4 is reduced, sarcomeres fail to be stably organized, and other sarcomeric proteins are degraded in response. Consistent with this idea, *tnnt2* RNA is present but Tnnt2 protein is diminished in 3-OST-7 morphants. We predicted that if Tpm4 serves as a lynchpin protein in 3-OST-7 function, then we should be able to rescue sarcomere organization and ventricular contraction in 3-OST-7 morphants by injection of *tpm4* RNA. Injection of *tpm4* RNA alone in control embryos had no perceived gross morphological effect, nor did it alter cardiac function (Figure S4). Strikingly, injection of *tpm4* RNA in 3-OST-7 morphants rescued ventricular contraction as assessed by looking at contraction in 48 hpf embryos (*p* = 0.0097, Figure S4) and by measuring ejection area (*p*<0.05, Figure 3L). In keeping with the rescued ventricular contraction, *tpm4* RNA injection in 3-OST-7 MO also rescued the organization and expression of sarcomeric proteins Tnnt2 and Tpm, and rescued the TEM appearance of sarcomeric structures in myofibrils (Figure 3I–3K). In contrast with the ability of *tpm4* RNA to rescue cardiac contraction in 3-OST-7 morphants, transient transgenic expression of *tnnt2* did not rescue cardiac contraction. As a control, embryos that were transgenic for a construct that drives cardiac expression of *tnnt2-IRES-EGFP* under the control of the *cmlc2* promoter had normal cardiac contraction. 3-OST-7 MO injected into this transgenic (scored by EGFP expression) resulted in decreased cardiac contraction, at frequencies comparable to 3-OST-7 MO in non-transgenic siblings (Figure S5). Together these results demonstrate that Tpm4 serves as a downstream lynchpin of 3-OST-7 function for normal cardiac ventricular contraction.

### Chamber Patterning, FGF, and Notch Signaling are Normal in 3-OST-7 Morphants

**Figure 4:**
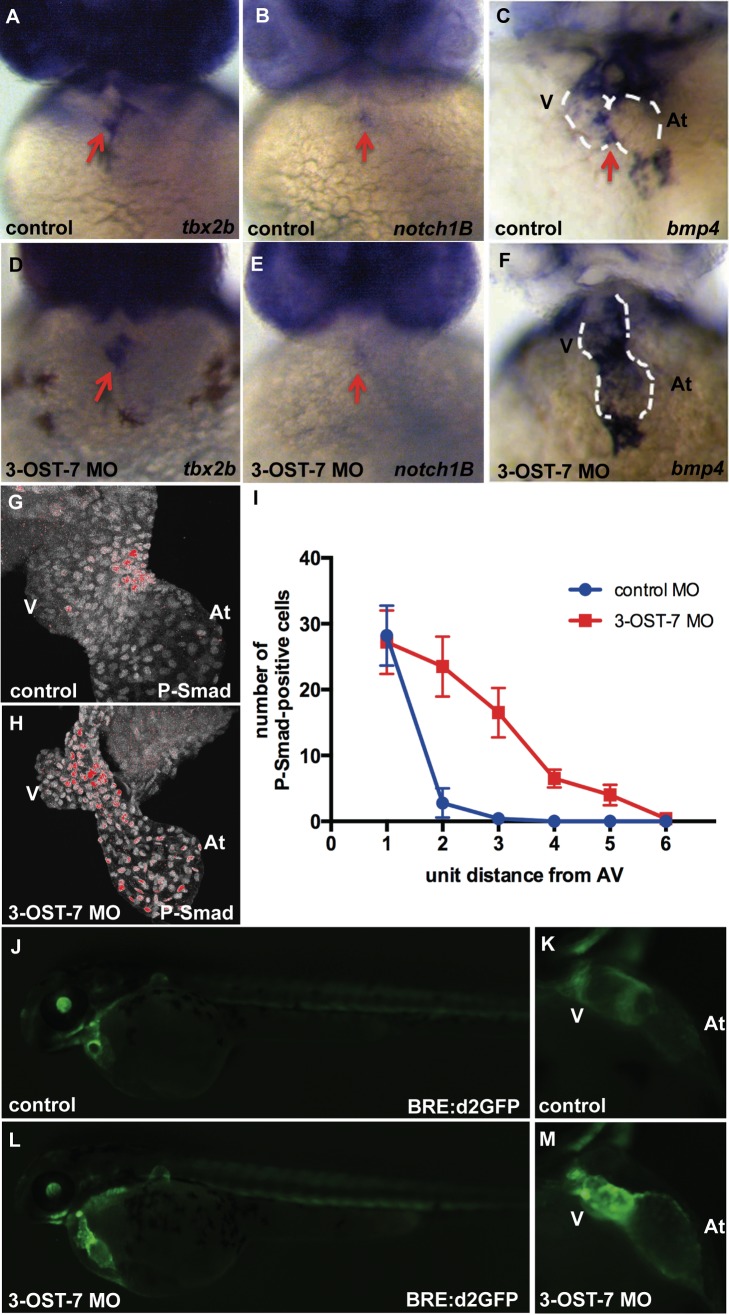
3-OST-7 controls region-specific BMP signaling in differentiating heart. ISH for *tbx2b* (A and D), *notch1B* (B and E) showed normal AV-restricted expression, whereas *bmp4* expression (C and F) showed ectopic expression in ventricular myocardium of 3-OST-7 morphants at 48 hpf (*n* = 30 for each group). IHC for P-Smad at 48 hpf showed delocalized expression in nuclei of 3-OST-7 morphant ventricle (H) compared to localized AV canal expression in control (G) (*n* = 10 for each group). (I) Graph depicting increased P-Smad-positive nuclei in the ventricle several unit distances away from the AV in 3-OST-7 morphants compared to P-Smad-positive nuclei localized in the AV for control (error bars, standard deviation). Imaging of live *Tg(BRE:d2GFP)* fish (J and L) showed GFP expression localized to the AV junction in control (K) and expanded expression in ventricle in morphant (M). V, ventricle; At, atrium; red arrows point to AV; white dashed lines outline the hearts.

In addition to myofibrillogenesis and onset of contraction, the cardiac maturation program involves a comprehensive patterning of myocardial cells into either contracting chamber myocardium (atrium or ventricle) or nonchamber, noncontracting myocardium (sinus venosus, atrioventricular or AV canal, and outflow tract) [28],[29]. To determine whether this patterning occurs in 3-OST-7 morphants, we performed ISH for *tbx2* (*tbx2b* in zebrafish) and *anf* in 48 hpf control embryos and 3-OST-7 morphants. *Tbx2b* is normally expressed in the AV canal (nonchamber myocardium) at 48 hpf [30], and we observed a similar pattern of expression in both control embryos and 3-OST-7 morphants (Figure 4A and 4D). Similarly, *anf*, which is normally expressed in atrium and ventricle (chamber myocardium) at 48 hpf, had a similar pattern of expression in 3-OST-7 morphants (Figure S7C and S7D). Together these results suggest that 3-OST-7 morphants undergo normal patterning and segregation of chamber and nonchamber myocardium.

We also investigated whether two major developmental signaling pathways, FGF and Notch signaling, are involved in 3-OST-7 regulation of ventricular contraction. FGF signaling was a strong candidate because it requires HSPG GAG chains for receptor-ligand complex formation [13],[31]–[33]. If loss of contraction caused by knockdown of 3-OST-7 occurs through deficient FGF signaling, direct perturbation of FGF signaling should mimic the noncontracting ventricle phenotype of 3-OST-7 morphants. However, FGF receptor 1 (*fgfr1*) knockdown resulted in ventricles that were smaller but had normal contractility (Figure S6A). Similarly, reducing or abolishing FGF signaling either in the zebrafish *fgf8/ace* mutant or by treatment with the FGFR inhibitor SU5402 also resulted in small hearts with particularly notable reductions of the ventricle, but no reported alterations in contraction [34],[35]. The normal cardiac contraction in FGF pathway manipulations suggests that FGF signaling is not a component of the 3-OST-7-dependent pathway.

In *Drosophila*, the Notch pathway is dependent on 3-O-sulfation by 3-OST-B [36]. More importantly, *deltaD*, a Notch ligand, was one of the most downregulated genes in the microarray analysis comparing control embryo hearts and 3-OST-7 morphant hearts at 48 hpf (7.0-fold decreased, *p* = 1.86×10^−2^). To determine whether 3-OST-7 regulates ventricular contraction by way of the Notch signaling pathway, we assessed whether the noncontracting ventricle phenotype is recapitulated in *deltaD/aei^AG49^* mutant embryos [37]. Embryos carrying a homozygous mutation in the *deltaD* gene were identified by misshapped somites posterior to the ninth somite [37] and separated from wild-type or heterozygous siblings at 18 hpf. The hearts were then scored for ventricular contraction at 48 hpf. Cardiac contraction was normal in *deltaD/aei^AG49^* mutants (*n* = 34 mutants; *n* = 94 wild-type siblings). Since DeltaD is one of four Delta ligands in zebrafish, it is possible that other Delta ligands might be compensating for loss of DeltaD in *deltaD/aei^AG49^* mutants. To more broadly block Notch signaling, we used DAPT, a γ-secretase inhibitor. Continuous treatment from 5 hpf, when cells are fated to become myocytes [38], to 48 hpf did not result in ventricular noncontraction at 48 hpf (Figure S6B), but disrupted somite formation, indicative of treatment efficacy. Treatments during narrower developmental windows gave similar results, with normal cardiac contraction (Figure S6B). Together these results suggest that Notch signaling is not a component of the 3-OST-7-dependent pathway for cardiac contraction.

### BMP Signaling Is Expanded in 3-OST-7 Morphants

B*mp4*, *versican*, and *notch1b* expression patterns are progressively restricted to the AV junction during cardiac development. All three genes are expressed along the antero-posterior length of the heart at 24 hpf and are subsequently restricted to the AV canal and excluded from expression in the maturing ventricle by 48 hpf (Figures 4B, 4C, and S7A), as previously reported [39]–[41]. However, in contrast to controls, in 3-OST-7 morphants *bmp4* (Figure 4F) and *versican* (Figure S7B) were ectopically expressed in ventricles at 48 hpf. *Bmp4* and *versican* remained ectopically expressed in ventricular myocytes at 3 days postfertilization (*n* = 40 embryos). In contrast to *bmp4* and *versican*, *notch1B* was expressed solely in the AV canal of 3-OST-7 morphants (Figure 4E), similar to control embryos, and *tie2* expression, assessed in *Tg(tie2:EGFP)* embryos, was expressed normally in the AV canal in both control and 3-OST-7 morphant embryos (Figure S7E and S7F). Normal *notch1B* and *tie2* expression suggest that the failure of *bmp4* and *versican* to become AV canal-restricted was not merely due to developmental delay, nor to an overall mispatterning of AV boundaries. Together these results indicate 3-OST-7 morphant hearts achieve normal AV boundary formation, but fail to exclude *bmp4* expression from ventricular myocytes.

To investigate whether ectopic expression of *bmp4* in the ventricle affects BMP signaling, we performed IHC for phosphorylated-Smad1/5/8 (P-Smad), a downstream marker for Bmp signaling. P-Smad was localized most strongly in the nuclei of AV canal cells in control hearts (Figure 4G), which corresponds to *bmp4* being normally restricted to that region at 48 hpf. In contrast, P-Smad was found strongly expressed in the nuclei of ventricular cells in 3-OST-7 morphant hearts as well as in nuclei of AV canal cells (Figure 4H), which corresponds to the ectopic expression of *bmp4* in the ventricle. In order to quantify this effect, we divided the ventricle images into six domains (domain 1 contains the AV junction and domain 6 was the region farthest from the AV junction) and counted the number of P-Smad-positive nuclei within each domain (Figure 4I). In 3-OST-7 morphants, an increased number of P-Smad-positive nuclei were present even in the domains farthest from the AV, while there were very few P-Smad positive nuclei outside the AV domain in control hearts. Interestingly, the number of P-Smad positive nuclei in 3-OST-7 morphants appears as a gradient, with the highest numbers close to the AV junction (Figure 4I). We also utilized the transgenic *Tg(BRE:d2GFP)* fish [42] to visualize dynamic transcriptional response to BMP signaling in live embryos. In control embryos, GFP expression in the heart was observed primarily in the AV junction (Figure 4J and 4K). In contrast, GFP expression was expanded to the whole ventricle in 3-OST-7 morphants (Figure 4L and 4M), in keeping with the expanded *bmp4* RNA expression and the expanded P-Smad nuclear staining. These results suggest that the role of 3-OST-7 is to confine *bmp4* expression and downstream BMP response to the AV junction, and to prevent BMP signaling from spreading into ventricular myocardium at 48 hpf.

To investigate whether other components of the BMP signaling pathway are involved in the 3-OST-7 morphant phenotype, we performed ISH analysis on nine BMP receptors at four different developmental timepoints (17 somite stage, 24 hpf, 36 hpf, and 48 hpf): *bmpr1aa* (*alk3a*), *bmpr1ab* (*alk3b*), *bmpr1ba* (*alk6a*), *bmpr1bb* (*alk6b*), *bmpr2b*, *acvr1l* (*alk8*), *acvr2aa* (*acvr2a*), *acvr2b*, and *acvrl1* (*alk1*) (Table S3). Of these, *bmpr2b* and *alk8* had altered heart expression in 3-OST-7 morphants, with strongly increased *bmpr2b* expression in the heart (Figure S8C) and *alk8* expression in the outflow tract (Figure S8D) that were not observed in controls (Figure S8A and S8B).

### Ectopic Ventricular Expression of *Bmp4* Occurs in Two Other Models of Noncontraction

**Figure 5:**
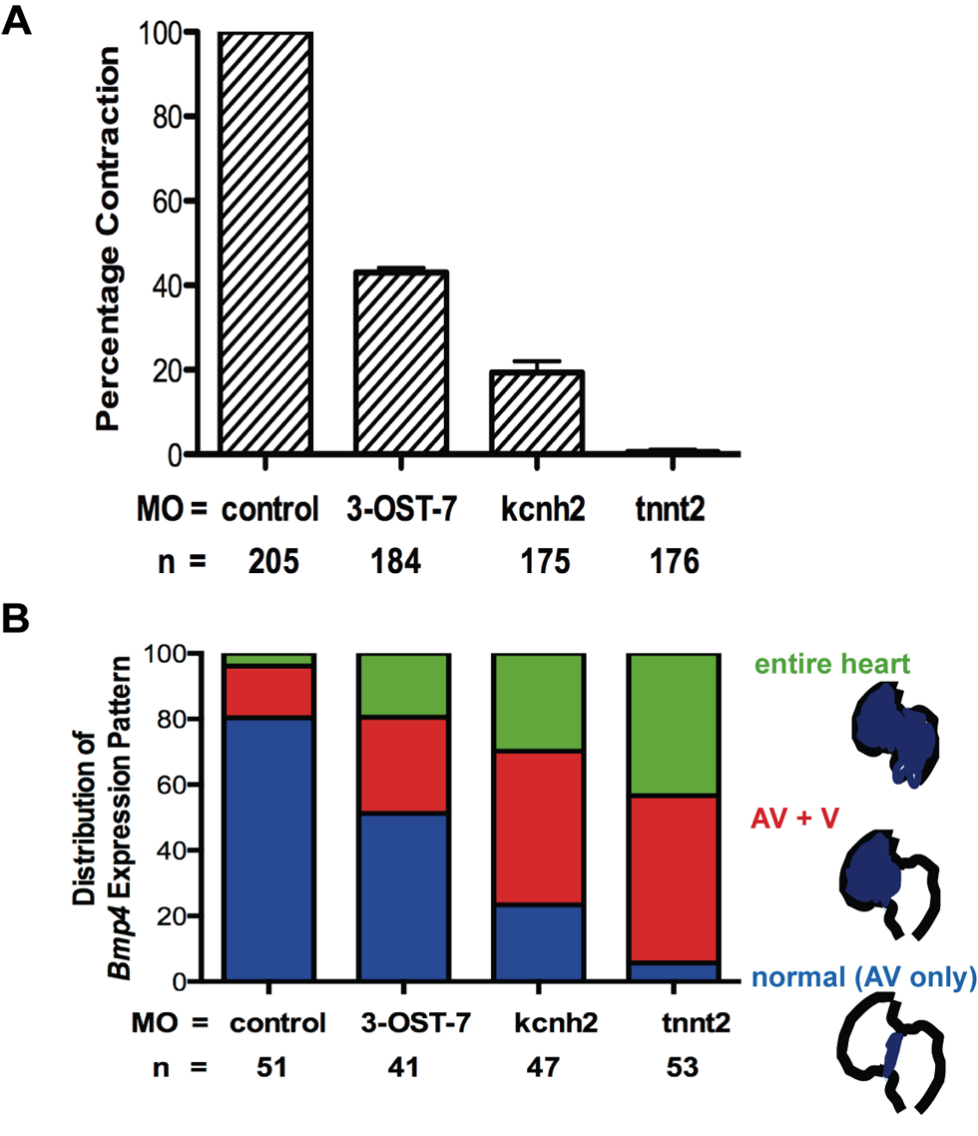
Noncontraction is correlated with ectopic *bmp4* expression. (A) Graph comparing the percentage of normal contraction with 3-OST-7, *kcnh2*, and *tnnt2* MO injections. Error bars, SEM (B) Graph comparing patterns of *bmp4* expression at 48 hpf among control embryos (injected with 3-OST-5 MO), 3-OST-7 morphants, *kchn2* morphants, and *tnnt2* morphants. Loss of contraction correlates with ectopic *bmp4* expression in the ventricle (AV+V) or throughout the entire heart in 3-OST-7, *kcnh2* and *tnnt2* morphants.

The expansion of BMP signaling into the ventricular myocardium in 3-OST-7 morphants provides a correlation between absence of ventricular contraction and expanded or ectopic expression of *bmp4* in ventricular myocardium. To determine whether this correlation occurs in other distinct pathways that lead to defective contraction, we examined *bmp4* expression in morphants of the potassium channel gene *kcnh2* and sarcomeric protein cardiac troponin T gene *tnnt2*. *Kcnh2* and *tnnt2* mutants both have “silent” (i.e., noncontracting) hearts and MO knockdown of these genes phenocopy the mutant phenotypes [24],[43], which we scored as percent of embryos with cardiac contraction defect (Figure 5A).

*Bmp4* expression is significantly expanded in the *kcnh2* and *tnnt2* morphants (Figure 5B), which we classified and scored in three categories: normal AV-restricted expression (AV only, blue, Figure 5B), ectopic expression expanded into ventricle (AV+V, red, Figure 5B), and ectopic expression expanded into both atrium and ventricle (entire heart, green, Figure 5B). Strikingly, only 23.4% and 5.7% of embryos had normal AV-restricted expression with injection of *kcnh2* and *tnnt2* MO, respectively (blue, Figure 5B). Most of the morphants (76.6% and 94.3% for *kcnh2* and *tnnt2* morphants, respectively) had expanded, ectopic expression of *bmp4* in the ventricle (red and green, Figure 5B).

Interestingly, in *kcnh2*, *tnnt2*, and 3-OST-7 morphants, the percentage of ectopic *bmp4* expression correlated with percentage of noncontraction (comparing Figure 5B and 5A). For example, knockdown of 3-OST-7 resulted in 56.9% of embryos having ventricular noncontraction (Figure 5A) and 48.8% had ectopic *bmp4* expression (red and green, Figure 5B), the least noncontraction and ectopic *bmp4* expression fractions observed among the three MO knockdowns. In contrast, knockdown of *tnnt2* resulted in 99.4% of embryos with noncontraction (Figure 5A), the highest noncontraction fraction among the three knockdowns, which correlated with the highest fraction of ectopic *bmp4* expression (red and green, Figure 5B). These results demonstrate that the correlation between noncontraction and ectopic *bmp4* expression is conserved in three very distinct models of defective cardiac contraction.

### 3-OST-7-Mediated Regulation of *Bmp4* Expression Is Required for Normal Contraction

**Figure 6:**
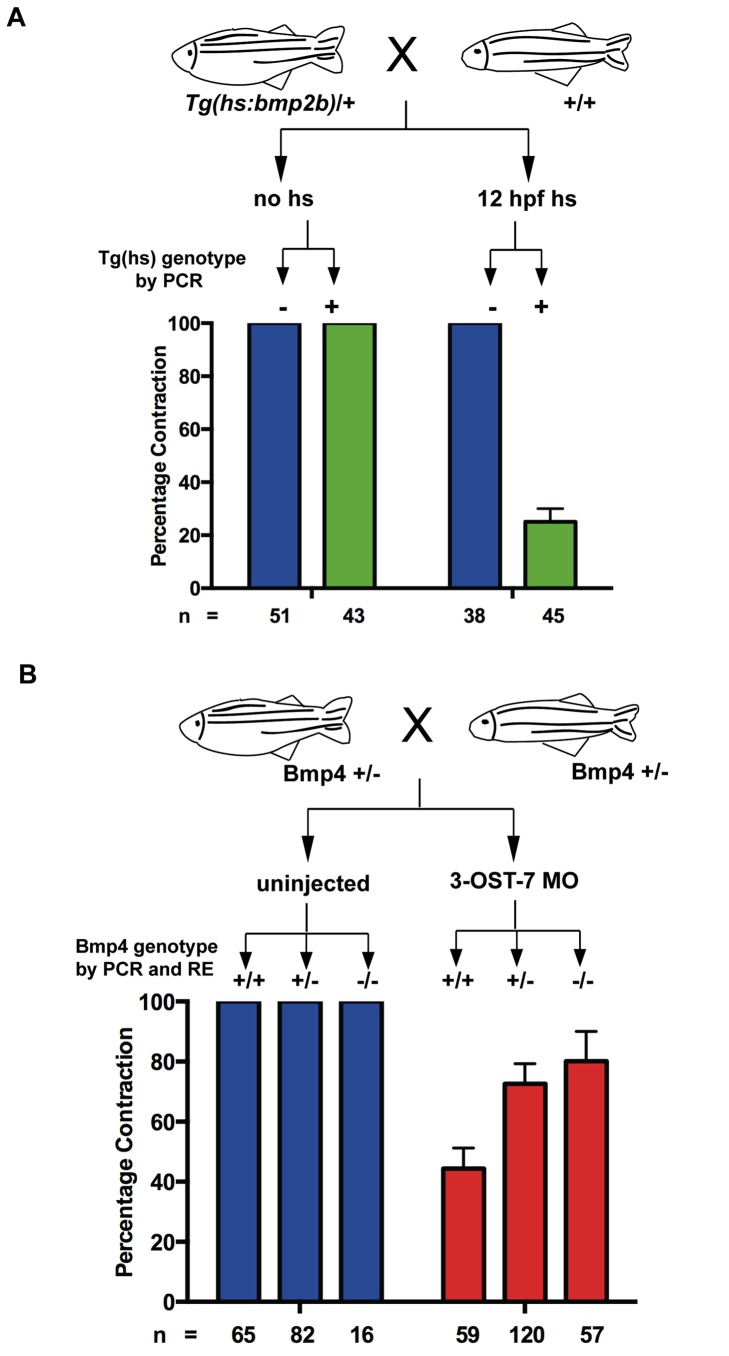
3-OST-7 regulates cardiac contraction by constraining BMP signaling. (A) *Tg(hs:bmp2b)* heterozygotes were crossed to wild-type zebrafish and embryos were either untreated (no hs) or heat-shocked at 12 hpf. Embryos in each group were scored for ventricular contraction, and then genotyped for presence of heat-shock transgene. Graph depicts percentage contraction of embryos with transgene (green) or without (blue) in each treatment group. Induction of BMP signaling led to ventricular noncontraction. (B) *bmp4^st72^* heterozygotes were crossed and embryos were either uninjected or injected with 3-OST-7 MO. Embryos in each group were scored for ventricular noncontraction, and then genotyped for *bmp4* mutation (RE, digestion with *SpeI*). Graph depicts percentage contraction of each genotypic class in uninjected embryos (blue) or embryos injected with 3-OST-7 MO (red). Ventricular noncontraction was rescued in 3-OST-7 morphants by *bmp4^st72^* mutation.

The above results indicate a correlation between expanded BMP expression in ventricular myocytes and failure to contract, but they do not address causality. Is expansion of BMP expression, as seen in 3-OST-7 morphants, capable of preventing ventricular contraction? To test the hypothesis that the ectopic expression of *bmp4* in the ventricle causes a noncontracting phenotype, we utilized the transgenic *Tg(hsp70:bmp2b)* zebrafish [44] and performed heat-shock to induce BMP signaling. We crossed heterozygous *Tg(hsp70:bmp2b)*/+ fish to wild-type AB and subjected half of the progeny to heat-shock (37°C for 30 min) at 12 hpf, while leaving the remaining half untreated (Figure 6A). We scored for ventricular noncontraction, and then confirmed presence of the heat-shock transgene by PCR. We found that heat-shock at 12 hpf (Figure 6A), but not at 24 hpf and 36 hpf (Table S4), resulted in ventricular noncontraction.

These results indicate that ectopic overexpression of BMP is capable of blocking ventricular contraction, but do not test whether the expanded expression of BMP observed in 3-OST-7 morphants is causative of the noncontracting phenotype. If the function of 3-OST-7 is to reduce or constrain BMP expression from ventricular myocytes, and excessive BMP is causative of contraction defects in 3-OST-7 morphants, then reduction of endogenous BMP levels in 3-OST-7 morphants should alleviate the ventricular contraction defect. To test this hypothesis, we utilized the zebrafish *bmp4^st72^* mutant [45] and asked whether genetic reduction of *bmp4* will rescue the noncontracting ventricle phenotype caused by knockdown of 3-OST-7. We injected 3-OST-7 MO into embryos from crosses between *bmp4^st72^/+* heterozygotes, with uninjected embryos from the same genetic crosses serving as control (Figure 6B). The embryos were then segregated by cardiac phenotype: normal hearts or noncontracting ventricles at 48 hpf. In some cases a slight AV morphological defect was also observed, as part of the *bmp4^st72^* mutant phenotype seen in both uninjected and injected embryos, and these were counted among the normally contracting hearts. The individual embryos were then genotyped. In uninjected embryos, all embryos displayed normal cardiac contraction, regardless of wild-type, heterozygous, or homozygous genotype for *bmp4^st72^* (Figure 6B), indicating that cardiac contraction was not affected in the absence of 3-OST-7 MO. In siblings that were genotypically wild-type for *bmp4* (*+/+*) and injected with 3-OST-7 MO, the percentage of embryos with normal ventricular contraction was only 47.5%, similar to the range seen in other 3-OST-7 MO experiments (Figure 6B). Strikingly, the percentage of embryos with normal cardiac contraction was increased for 3-OST-7 MO injected *bmp4^st72^* mutants (*−/−*), with 73.7% of these mutants having a contracting ventricle (Figure 6B). Genomic DNA sequencing of the individual injected *bmp4^st72^* mutants confirmed that the 3-OST-7 MO targeted sequence was correct in *bmp4^st72^* mutants. These results indicate that reduction of endogenous BMP signaling is capable of rescuing ventricular contraction in 3-OST-7 morphants. Combined with the observation that ectopic BMP signaling can cause a noncontracting ventricle phenotype, these results indicate that 3-OST-7 functions to constrain BMP signaling to the AV junction and to reduce BMP signaling in the ventricle, thereby allowing normal cardiac ventricular contraction.

## Discussion

In this study we demonstrate that 3-OST-7, one of the enzymes that places a rare 3-O-sulfation on GAG chains on HSPGs, has a novel and highly specific function in cardiac development. In 3-OST-7 knockdown zebrafish, early cardiac cell specification, patterning, cardiac tube looping, and cardiomyocyte electrophysiology are normal, but ventricle contraction is defective. We show that 3-OST-7 is required for the normal accumulation of *tpm4* mRNA in the ventricle. Tpm4 protein appears to be a lynchpin in ventricular sarcomere assembly and stabilization, because overexpression of Tpm4 protein by *tpm4* mRNA injection in 3-OST-7 morphants rescues the levels and organization of other sarcomeric proteins, rescues sarcomere structure, and rescues ventricular contraction. Tpm4 is also reduced in the atrium in 3-OST-7 morphants, but either this level of reduction is not sufficient to affect atrial contraction or some other contractile component might compensate functionally for diminished Tpm4 in the atrium. In contrast, transgenic overexpression of cardiac troponin T *tnnt2* cannot rescue cardiac contraction in 3-OST-7 morphants. Thus, knockdown of 3-OST-7 uncouples contraction from the normally functioning excitation cycle by perturbing *tpm4* mRNA accumulation, leading to defective myofibrillogenesis. This places the 3-OST-7-dependent 3-O-sulfation of extracellular GAG chains as the first member of an otherwise unknown signaling pathway that is upstream of *tpm4* regulation and coordinated sarcomere assembly.

**Figure 7:**
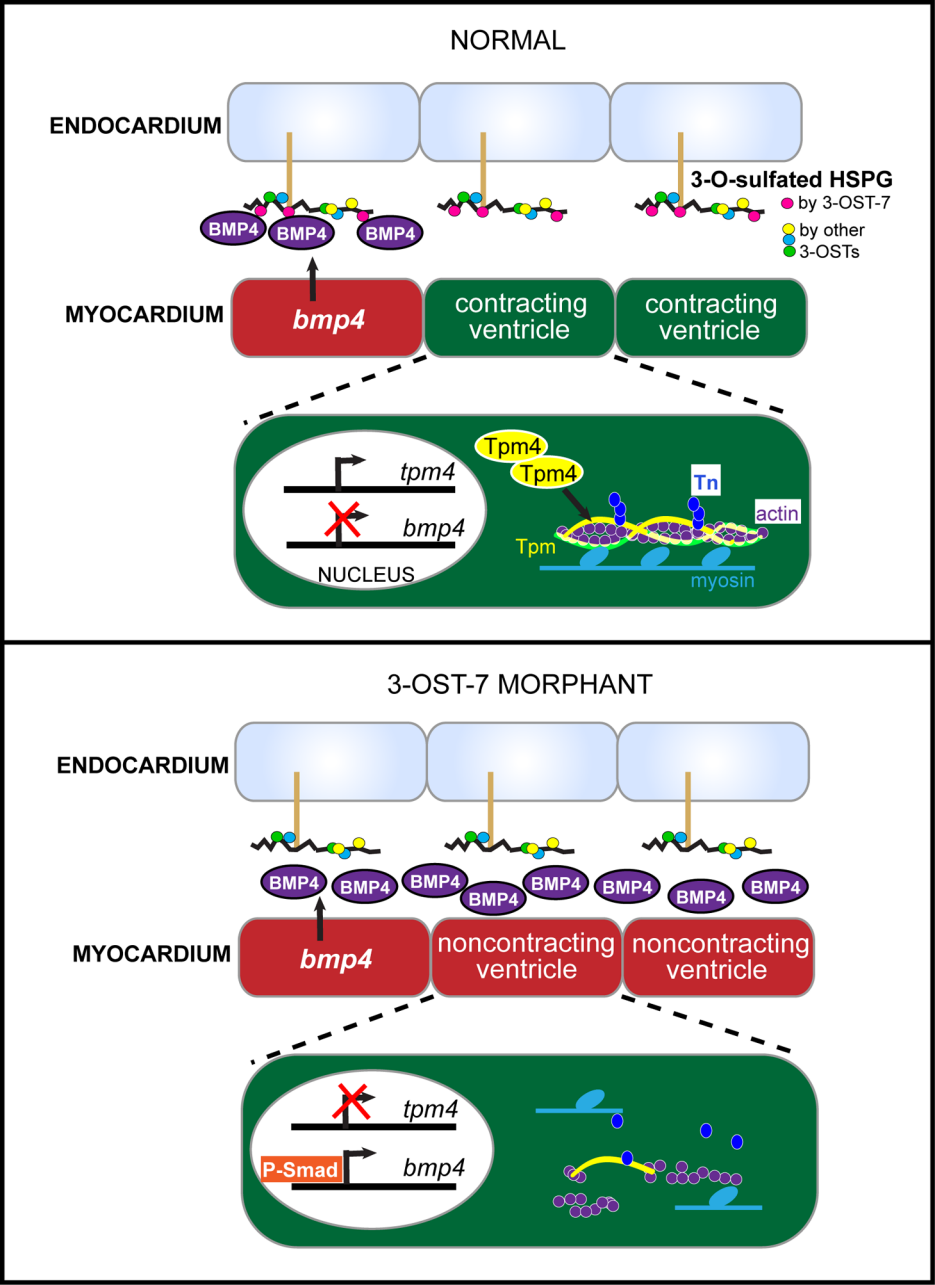
Model for role of 3-O-sulfation catalyzed by 3-OST-7 in cardiac development. Under normal conditions, specific 3-OST-7-dependent 3-O-sulfation patterns (pink circles) on endocardial HSPGs constrain *bmp4* in nonchamber (noncontracting) myocardium (AV junction, red compartment), allowing transcription of *tpm4* in contracting myocardium (ventricle, green compartment). Tpm4 then stabilizes the sarcomere and ensures proper contraction (Tn, troponin). Knockdown of 3-OST-7 results in loss of 3-O-sulfation, expansion of *bmp4* and BMP signaling and P-Smad delocalization into ventricular myocardium. High levels of BMP signaling lead to reduced levels of *tpm4* transcripts and Tpm4 proteins, which then disrupt sarcomere assembly and lead to noncontraction.

We propose that 3-OST-7 functions in the endocardium by modifying HSPGs at the interface between endocardium and myocardium in order to constrain BMP signaling to the AV junction and dampen BMP signaling in functional myocardium (Figure 7). The cardiac ventricular contraction defect in 3-OST-7 morphants could be rescued by ubiquitous transgenic expression of 3-OST-7 and by lineage-specific expression in the endocardium, but surprisingly not by lineage specific expression in the myocardium. This would suggest 3-O-sulfation of HSPGs by 3-OST-7 mediates cell-cell communication between myocardium and endocardium to regulate *tpm4* transcription (Figure 7). In the presence of normal 3-OST-7 function, BMP signaling is constrained to the AV junction and precluded from functional myocardium, as reflected in high levels of P-Smad in nuclei in the AV junction and little or no P-Smad in adjacent functional cardiomyocytes. Since 3-OST-7 appears to be ubiquitously expressed, the spatial regulation of BMP4 signaling is likely due to positive feedback loops within the BMP pathway that are constrained by 3-OST-7 function. Other studies support the idea of positive feedback loops, showing that ectopic BMP expression activates endogenous BMP expression in *Xenopus* embryos, and correspondingly, loss of BMP ligands *swirl* (*bmp2b*), *somitabun* (*smad5*), or *snailhouse* (*bmp7*) in zebrafish mutants results in loss of *bmp2b* expression [46]–[49]. We do not know whether this constraint on BMP signaling occurs by direct interaction of BMP4 and/or its receptors with 3-OST-7 modified HSPGs, or indirectly through other pathways, but it would be exciting in future studies to assess if BMP4 directly bind to specifically modified, 3-O-sulfated HSPGs. The constraint of BMP signaling allows functional cardiomyocytes to accumulate normal levels of *tpm4* mRNA and Tpm4 protein, which then serves as a lynchpin for the organization of normal contractile apparatus. The importance of BMP regulation is evident both from the ability of excessive BMP signaling to block cardiac contraction and the ability of reduced BMP levels to rescue contraction in 3-OST-7 MO. In the absence of 3-OST-7 function, the normal endogenous BMP signaling that occurs in the AV junction at 48 hpf spreads ectopically into myocardium beyond its normal boundaries in the AV junction, most likely mediated by the BMP receptor BMPR2B, which we show to be ectopically expressed in 3-OST-7 morphant hearts. This results in high levels of P-Smad in the nucleus of ventricular myocytes. High levels of BMP signaling result in reduced levels of *tpm4* mRNA, thereby removing the Tpm4 lynchpin and leading to failure of contractile apparatus organization. It is not known whether the reduction of tpm4 mRNA is due to direct transcriptional suppression by the increased levels of P-Smad in the ventricular nuclei, or to indirect effects. Thus, although ventricular myocytes have normal cycling calcium and electrophysiology, they are incapable of contracting. Interestingly, other models of cardiac noncontraction (*kcnh2* and *tnnt2* morphants) also display expanded expression of *bmp4*, suggesting there might be an inappropriate positive feedback loop between overexpression of BMP and a failure of cardiomyocytes to contract. We would not expect manipulations of the BMP4 pathway to rescue ventricular noncontraction in the *kcnh2* and *tnnt2* morphants or mutants, since other critical components, of either the excitation-contraction coupling process or contractile machinery, are still missing. It is interesting to note that zebrafish *tbx5* [50], *apc* [51], *foxn4* [30], and *tmem2* [52],[53] mutants that have a similar expansion of *bmp4* expression in the ventricle also have poor contractility, although the noncontraction phenotype in these mutants appears to be less penetrant and have a later onset than 48 hpf. These genes have been shown to control AV canal formation, and experiments in the *tmem2* mutants have shown that expanded *bmp4* expression facilitates expansion of the AV canal markers *hyaluronan synthase 2* and Alcama [53], suggesting an expansion of noncontracting, nonchamber myocardium. Our results uncover a unique role for *bmp4* in promoting a noncontracting, nonchamber myocardium in that other markers that distinguish between chamber and nonchamber myocardium were normal (*tbx2*, *anf*, and *notch1b*). The ability of *bmp4* to drive myocardium toward noncontracting, nonchamber myocardium is constrained by 3-O-sulfation function (Figure 7).

It is striking that the regulation of BMP signaling can be controlled by a rare modification of 3-O-sulfation on HSPGs, and that loss of this regulation has dramatic effects on the ability of the heart to function. Even more striking is that other 3-OST family members, many of which are expressed ubiquitously in these early stages of development [16], do not compensate for the loss of 3-OST-7. Knockdown of other 3-OST family members have distinct phenotypes and regulate other cell signaling pathways, including FGF signaling [18], but do not have the cardiac ventricular contraction defect described here for 3-OST-7. The regulation of ligand gradients and signaling by HSPG modification enzymes has been shown in *Drosophila* [11],[14], which has a limited number of genes for each step in the pathway. This level of precise regulation has not been described in vertebrates, in which each enzymatic step has large number of family members. Here, our results suggest that 3-OST-7 has a unique ability to generate a distinct modification on GAG chains of HSPGs, and this modification is necessary for the spatial regulation of BMP signaling during cardiac development, necessary for ventricle contraction.

## Materials and Methods

### Zebrafish Lines and Ethics Statement

All zebrafish experiments were performed in accordance to protocols approved by IACUC. Zebrafish were maintained under standard laboratory conditions at 28.5°C. In addition to Oregon AB wild-type, the following transgenic and mutant lines were used: *Tg(cmlc2:GFP)* [17], *(Tg(cmlc2-DsRed-nuc)* [19], *Tg(fli1:EGFP)* [22], *Tg(cmcl2:gCaMP)^s878^* [26], *Tg(hsp70:bmp2b)* [44], *Tg(BRE:d2GFP)* [42], *bmp4^st72^* [45], and *deltaD/aei^AG49^* [37].

### Morpholino Injection

MO oligonucleotides were obtained from Gene Tools, LLC. The following sequences and concentrations were used: translation-blocking 3-OST-7 MO1, 5′-CACATAACTCAGAAGATTGGCCATG-3′, 5.4 ng; splice-blocking 3-OST-7 MO2, 5′- CACATCTGGAAGACACAAGAGAGAG-3′, 1.8 ng; 3-OST-5 MO, 5′-GTCCAGTCAGGTCAAGGGCAGCTCA-3′, 2.7 ng; 3-OST-3Z MO, 5′-GTCCAGTCAGGTCAAGGGCAGCTCA-3′, 5.4 ng; translation-blocking *kcnh2* MO [24], 2.3 ng; translation-blocking *tnnt2* MO [43], 4 ng; translation-blocking *fgfr1* MO1 [54], 4 ng; and translation-blocking *fgfr1* MO2 [54], 8 ng. Embryos were injected at the 1–2 cell stage.

### Transgenesis and mRNA Rescue

The Tol2kit cloning system was used to generate *Tg(β-actin:3-OST-7-IEP)*, *Tg(cmlc2:3-OST-7-IEP)*, and *Tg(fli1:3-OST-7-IEP)*. Multisite recombination reactions were performed as previously described [20]. Transposase RNA was synthesized using mMessage mMachine kit (Ambion). 25 pg of transposase RNA and 30 pg of *β-actin:3-OST-7-IEP*, *cmlc2:3-OST-7-IEP* or *fli1:3-OST-7-IEP* plasmid DNA were injected into wild-type AB fish at the one-cell stage. Potential transgenic founders (TF) were identified by scoring for GFP expression in hearts (*cmlc2:3-OST-7-IEP* and *fli1:3-OST-7-IEP*) or ubiquitous GFP expression (*β-actin:3-OST-7-IEP*). Potential TFs were then crossed to wild-type AB fish to check for GFP expression. Those that gave GFP-positive transgenic embryos were subsequently used for rescue experiments where 3-OST-7 MO2 was injected into embryos from TF×AB matings. At 48 hpf, embryos were sorted by GFP fluorescence, then scored for ventricular noncontraction.

For *tpm4* rescue experiments, *tpm4* RNA was synthesized using the mMessage mMachine kit (Ambion) from the linearized pXT7-tpm4-tv1 expression vector [55]. 175 pg of RNA was co-injected with 5.4 ng of 3-OST-7 MO1 at the one-cell stage.

For *tnnt2* rescue experiments, 25 pg of transposase RNA, 30 pg of *cmlc2:tnnt2-IRES-EGFP* [56], and 5.4 ng 3-OST-7 MO1 were injected into wild-type AB fish at the one-cell stage. Those that gave GFP-positive transgenic embryos were scored for rescue of ventricular noncontraction.

### Data Acquisition and Processing for 3D Myocyte Reconstruction

48 hpf explanted hearts were placed in physiological solution containing 0 mM Ca^2+^ and 10 µM blebbistatin. The sarcolemma was labeled using wheat germ agglutinin conjugated to Alexa Fluor 555 (Invitrogen). Using a confocal microscope (LSM 5 Live Duo, Carl Zeiss) equipped with a 40× oil immersion lens, samples were excited with a 543 nm laser and emission collected with a long-pass 560 nm filter. Image stacks were acquired with a resolution of 0.2 µm×0.2 µm×0.2 µm. Correction of depth-dependent attenuation, deconvolution, and 3D reconstruction of confocal images were performed as previously described [57].

### *In Situ* Hybridization

Digoxigenin-labeled antisense riboprobes were synthesized using Digoxigenin RNA Labeling Kit (Roche). cDNA plasmids encoding *hand2*, *nkx2.5*, *cmlc2*, *amhc*, *vmhc*, *tnnt2*, *tpm4* [55], *bmp4*, *versican*, *notch1B*, *anf*, *tbx2b*, *bmpr1aa* (*alk3a*), *bmpr1ab* (*alk3b*), *bmpr1ba* (*alk6a*), *bmpr1bb* (*alk6b*), *bmpr2b*, *acvr1l* (*alk8*), *acvr2aa* (*acvr2a*), *acvr2b*, *acvrl1* (*alk1*) [58], *sdc2*, *sdc3*, and *sdc4* were used. ISH were performed as previously described [59], with anti-digoxigenin antibody incubation carried out using a Biolane HTI machine. Embryos were cleared in 70% glycerol and photographed with a Nikon SMZ1000 camera. Digital images were processed with Adobe Photoshop CS4.

### Calcium Transient Recording

Ca^2+^ transients were recorded as previously described [60]. Fluorescent signals (F) were normalized to baseline values (F_o_). The maximum Ca^2+^ transient amplitude (F_Max_/F_o_) was determined by averaging the peak amplitude of three consecutive transient signals. The decay of the calcium transient was determined by a monoexponential fit of the decaying signal and averaging value of three consecutive transient signals.

### Optical Mapping

48 hpf zebrafish was placed on a coverglass. Electromechanical isolation was achieved with 2,3-BDM (Sigma) at 10 mmol/l applied 15 minutes before imaging. Single plane widefield epifluorescence images of the heart were obtained with a Nikon TE-2000 inverted microscope using a 40× Plan Apo air objective, Xcite-120 (Exfo) widefield epifluorescent source and standard FITC filter set. Images were acquired with a Coolsnap HQ camera (Photometrics) using Metavue software (Molecular Devices) in stream acquisition mode at a frame rate of 30 ms/frame (512×512 pixels). Image processing consisted first of manual adjustment of minor spatial shifts of the image over a temporal imaging series. Then, the fluorescence intensity of each pixel in a 2D map was normalized to its percentage between the minimum and maximum recorded values of the pixel over the full series. Isochronal lines at 20 ms intervals were obtained by identifying the maximal spatial gradient for a given time point. The color-coded scheme in each panel and video describes progressive activation of the heart with white/red cells and black/blue cells indicating depolarization and repolarization, respectively. Software processing was performed with Metavue software and procedures written in MATLAB (MathWorks).

### Immunohistochemistry

IHC using the primary antibodies MF20 (Developmental Studies Hybridoma Bank, 1∶10), CT3 (Developmental Studies Hybridoma Bank, 1∶10), CH1 (Developmental Studies Hybridoma Bank, 1∶10), and P-Smad1/5/8 (Cell Signaling Technology, 1∶100) was performed as previously described [54]. Secondary antibody, either donkey anti-mouse AlexaFluor488 (Molecular Probes) or goat anti-mouse AlexaFluor488 (Molecular Probes), was used in 1∶100 dilution. Images were acquired using an Olympus Fluoview FV300 laser scanning confocal microscope. Digital images were processed with Adobe Photoshop CS4.

### Rescue in *Bmp4^st72^* Embryos

Embryos from *bmp4^st72^/+*×*bmp4^st72^/+* matings were injected with 3-OST-7 MO. At 48 hpf, the hearts were scored for noncontracting ventricle or wild-type phenotype. Genomic DNA was extracted from each individual embryo and genotyped using the following dCAPS primers, 5′-TGGTGAGGCACAACACCTCCAACTAG-3′ (forward) and 5′-CCGAGTCAGCGGGTGACTTTTGCCGTC-3′ (reverse). The PCR products were digested with *SpeI* (NEB) and ran in 3% agarose gel. Digestion with *SpeI* releases 250 bp band in wild-type, 230 bp band in mutant, and both in heterozygotes. DNA genotyped to be from mutants were sequenced to verify compatibility with 3-OST-7 MO.

### Statistics

Statistical significance was analyzed using Student's *t*-test. Analysis was performed using GraphPad Prism (version 6.00 for Mac GraphPad Software). Results are considered significant when *p*<0.05 and results are expressed as mean ± standard error of the mean (SEM).

## Supporting Information

Figure S1 **Heart field specification proceeds normally in 3-OST-7 morphants.** Dorsal views (anterior on top) of control (uninjected, wild-type) (A, C, E, and K), control MO (injected with control 3-OST-3Z MO) (G and I), and 3-OST-7 morphant (B, D, F, H, J and L) embryos; *n* = 35 for each group. *ISH*s for: lateral plate mesoderm marker *hand2* (A and B, 17 hpf) and cardiac precursor cell marker *nkx2.5* (C and D, 17 hpf), myocardial precursor cell marker *cmlc2* (E and F, 17 hpf), atrial precursor cell marker *amhc* (I and J, 20 hpf), and ventricular precursor cell marker *vmhc* (K and L, 18 hpf) showed comparable levels and patterns of expression in control and 3-OST-7 morphant embryos. Imaging of *fli1* expression in *Tg(fli1:EGFP)* zebrafish at 18 hpf revealed endocardial lineage is intact in 3-OST-7 morphant embryos (G and H).
(TIF)

Figure S2 **Knockdown of 3-OST-7 disrupts sarcomere organization.** Whole mount IHC using anti-myosin (MF20) and phalloidin revealed myosin and actin filaments were disorganized in ventricles of 3-OST-7 morphants (B and D) compared with control (A and C) (*n* = 30 for each group). At, atrium; V, ventricle.
(TIF)

Figure S3 **3-OST-7 controls transcript levels of tpm4 but not those of tnnt2.** *In situ* analysis for *tnnt2* showed comparable transcript levels and patterns of expression for 3-OST-7 morphants (B, F, and J) and control (injected with control 3-OST-3Z MO) embryos (A, E, and I) at 20 hpf (A and B), 24 hpf (E and F), and 48 hpf (I and J). In contrast, *tpm4* transcripts were decreased in 3-OST-7 morphants (D and H) compared to control embryos (C and G) at 20 hpf (C and D) and 24 hpf (G and H). (A–D) are dorsal views with anterior on top; (E–J) are ventral views with anterior on top; *n* = 40 for each group. At, atrium; V, ventricle.
(TIF)

Figure S4 **Overexpression of** ***tpm4*** **rescues the noncontracting ventricle phenotype in 3-OST-7 morphant embryos.** Overexpression of *tpm4* in control embryos did not alter cardiac function. Strikingly, overexpression in 3-OST-7 morphants rescued ventricular noncontraction (*p* = 0.0097).
(TIFF)

Figure S5 **Overexpression of** ***tnnt2*** **using transient** ***cmlc2:tnnt2-IRES-EGFP*** **plasmid expression does not rescue the noncontracting ventricle phenotype in 3-OST-7 morphant embryos.** *Tnnt2* transgene expression was scored by EGFP expression. Injection of plasmid alone (blue bar) did not perturb ventricular contraction similar to control (white bar). Injection of both plasmid and 3-OST-7 MO (green bar) resulted in ventricular noncontraction at a percentage similar to injection of 3-OST-7 MO alone (red bar) (NS, *p* = 0.69).
(TIFF)

Figure S6 **Disrupting the FGF and Notch signaling pathways do not phenocopy the noncontracting ventricle phenotype of 3-OST-7 knockdown.** (A) Table showing percentage of normal contraction and small ventricles in control (uninjected) embryos, embryos injected with 4 ng *fgfr1* MO1, and embryos injected with 8 ng *fgfr1* MO2 at 48 hpf. Ventricular contraction appeared normal in all groups. (B) Timeline showing the time and duration of 75 µm DAPT treatment and table showing percentage of embryos with normal contraction. Control embryos were treated with 0.3% (v/v) DMSO. Ventricular contraction appeared normal in all treatments. To ensure DAPT was working, embryos treated with DAPT starting at 5 hpf were observed at 18 hpf for somite defects. All embryos that received DAPT treatment starting at this timepoint developed somite defects at 18 hpf. No somite disruption was observed in corresponding DMSO treatments.
(TIF)

Figure S7 **Knockdown of 3-OST-7 affects expression pattern of** ***versican*** **but does not alter expression patterns of other heart differentiation markers at 48 hpf.** *ISH* for *versican*, an AV myocardium-localized marker, showed ectopic expression in ventricular myocardium of 3-OST-7 morphants (A and B). Expression of *anf*, a marker for chamber myocardium, was comparable between control and 3-OST-7 morphants (C and D). *Tie2* expression, assessed in *Tg(tie2:EGFP)* embryos, was normally expressed in 3-OST-7 morphant AV endocardium (F) and is similar to control (E). Control groups (A and C) were injected with control 3-OST-3Z MO, control (E) was uninjected. V, ventricle; At, atrium; red arrows point to AV; dashed white lines outline the heart.
(TIF)

Figure S8 **Knockdown of 3-OST-7 alters expression patterns of BMP receptors** ***bmpr2b*** **and** ***alk8*** **.** *ISH* for *bmpr2b* (A, C) showed ectopic expression in heart (C, red arrow) of 3-OST-7 morphant. *ISH* for *alk8* (B, D) showed ectopic expression in outflow tract (D, red arrow) of 3-OST-7 morphant.
(TIF)

Table S1 **Action potential parameters recorded from control (uninjected, wild-type) embryos and 3-OST-7 morphants.**
(DOCX)

Table S2 **Statistical** ***t*** **-test comparison of action potential parameters between control (uninjected, wild-type) embryos and 3-OST-7 morphants.**
(DOCX)

Table S3 ***ISH*** **analysis comparing expression of nine BMP receptors in control wild-type and 3-OST-7 morphants.**
(DOCX)

Table S4 **Heat-shock of embryos from Tg(hs:bmp2b)/+×wild-type cross at 24 hpf and 36 hpf.**
(DOCX)

Video S1 **Lateral view of beating heart in control (uninjected, wild-type)** ***Tg(cmlc2:gfp)*** **embryo at 48 hpf.** Ventricle is left, atrium is right.
(MOV)

Video S2 **Lateral view of impaired ventricular contraction in 3-OST-7 morphant** ***Tg(cmlc2:gfp)*** **embryo at 48 hpf.** Ventricle is left, atrium is right.
(MOV)

Video S3 **Normal circulation in the trunk of control (uninjected, wild-type) embryo.**
(MOV)

Video S4 **Poor blood circulation in the trunk of 3-OST-7 morphant embryo.**
(MOV)

Video S5 **Calcium waves in explanted heart stained with Fluo-4 of control (injected with control 3-OST-5 MO) embryo.** Ventricle is right, atrium is left.
(MOV)

Video S6 **Calcium waves in explanted heart stained with Fluo-4 of 3-OST-7 morphant embryo.** Ventricle is right, atrium is left.
(MOV)

Video S7 **Calcium activation and progression of conduction in control (injected with control 3-OST-3Z MO)** ***Tg(cmlc2:gCaMP)^s878^*** **embryo.** Atrium is on top.
(MOV)

Video S8 **Calcium activation and progression of conduction in 3-OST-7 morphant** ***Tg(cmlc2:gCaMP)^s878^*** **embryo.** Atrium is slightly out of focus on top.
(MOV)

## Abbreviations

3-OST: 3-O-sulfotransferase
AV: atrioventricular
ECM: extracellular matrix
GAG: glycosaminoglycan
hpf: hours postfertilization
HSPG: heparan sulfate proteoglycan
IHC: immunohistochemistry
ISH: in situ hybridization
MO: morpholino
SEM: standard error of the mean
TEM: transmission electron microscopy

## References

[1] Shi Y, Katsev S, Cai C, Evans S (2000) BMP signaling is required for heart formation in vertebrates. Dev Biol 224: 226–237. 10926762 10.1006/dbio.2000.9802

[2] Walters MJ, Wayman GA, Christian JL (2001) Bone morphogenetic protein function is required for terminal differentiation of the heart but not for early expression of cardiac marker genes. Mech Dev 100: 263–273. 11165483 10.1016/s0925-4773(00)00535-9

[3] Yang L, Cai CL, Lin L, Qyang Y, Chung C, et al (2006) Isl1Cre reveals a common Bmp pathway in heart and limb development. Development 133: 1575–1585. 16556916 10.1242/dev.02322 PMC5576437

[4] Mandel EM, Kaltenbrun E, Callis TE, Zeng XX, Marques SR, et al (2010) The BMP pathway acts to directly regulate Tbx20 in the developing heart. Development 137: 1919–1929. 20460370 10.1242/dev.043588 PMC2867324

[5] Hoogaars WM, Barnett P, Moorman AF, Christoffels VM (2007) T-box factors determine cardiac design. Cell Mol Life Sci 64: 646–660. 17380306 10.1007/s00018-007-6518-z PMC2778635

[6] Rottbauer W, Baker K, Wo ZG, Mohideen MA, Cantiello HF, et al (2001) Growth and function of the embryonic heart depend upon the cardiac-specific L-type calcium channel alpha1 subunit. Dev Cell 1: 265–275. 11702785 10.1016/s1534-5807(01)00023-5

[7] Xu X, Meiler SE, Zhong TP, Mohideen M, Crossley DA, et al (2002) Cardiomyopathy in zebrafish due to mutation in an alternatively spliced exon of titin. Nat Genet 30: 205–209. 11788825 10.1038/ng816

[8] Berdougo E, Coleman H, Lee DH, Stainier DY, Yelon D (2003) Mutation of weak atrium/atrial myosin heavy chain disrupts atrial function and influences ventricular morphogenesis in zebrafish. Development 130: 6121–6129. 14573521 10.1242/dev.00838

[9] Huang C, Sheikh F, Hollander M, Cai C, Becker D, et al (2003) Embryonic atrial function is essential for mouse embryogenesis, cardiac morphogenesis and angiogenesis. Development 130: 6111–6119. 14573518 10.1242/dev.00831

[10] Park PW, Reizes O, Bernfield M (2000) Cell surface heparan sulfate proteoglycans: selective regulators of ligand-receptor encounters. J Biol Chem 275: 29923–29926. 10931855 10.1074/jbc.R000008200

[11] Hacker U, Nybakken K, Perrimon N (2005) Heparan sulphate proteoglycans: the sweet side of development. Nat Rev Mol Cell Biol 6: 530–541. 16072037 10.1038/nrm1681

[12] Lamanna WC, Kalus I, Padva M, Baldwin RJ, Merry CL, et al (2007) The heparanome–the enigma of encoding and decoding heparan sulfate sulfation. J Biotechnol 129: 290–307. 17337080 10.1016/j.jbiotec.2007.01.022

[13] Lin X (2004) Functions of heparan sulfate proteoglycans in cell signaling during development. Development 131: 6009–6021. 15563523 10.1242/dev.01522

[14] Bulow HE, Hobert O (2006) The molecular diversity of glycosaminoglycans shapes animal development. Annu Rev Cell Dev Biol 22: 375–407. 16805665 10.1146/annurev.cellbio.22.010605.093433

[15] Esko JD, Selleck SB (2002) Order out of chaos: assembly of ligand binding sites in heparan sulfate. Annu Rev Biochem 71: 435–471. 12045103 10.1146/annurev.biochem.71.110601.135458

[16] Cadwallader AB, Yost HJ (2006) Combinatorial expression patterns of heparan sulfate sulfotransferases in zebrafish: I. The 3-O-sulfotransferase family. Dev Dyn 235: 3423–3431. 17075882 10.1002/dvdy.20991

[17] Huang CJ, Tu CT, Hsiao CD, Hsieh FJ, Tsai HJ (2003) Germ-line transmission of a myocardium-specific GFP transgene reveals critical regulatory elements in the cardiac myosin light chain 2 promoter of zebrafish. Dev Dyn 228: 30–40. 12950077 10.1002/dvdy.10356

[18] Neugebauer JM, Cadwallader AB, Amack JD, Bisgrove BW, Yost HJ (2013) Differential roles for 3-OSTs in the regulation of cilia length and motility. Development 140: 3892–3902. 23946439 10.1242/dev.096388 PMC3754482

[19] Mably JD, Mohideen MA, Burns CG, Chen JN, Fishman MC (2003) heart of glass regulates the concentric growth of the heart in zebrafish. Curr Biol 13: 2138–2147. 14680629 10.1016/j.cub.2003.11.055

[20] Kwan KM, Fujimoto E, Grabher C, Mangum BD, Hardy ME, et al (2007) The Tol2kit: a multisite gateway-based construction kit for Tol2 transposon transgenesis constructs. Dev Dyn 236: 3088–3099. 17937395 10.1002/dvdy.21343

[21] Schoenebeck JJ, Keegan BR, Yelon D (2007) Vessel and blood specification override cardiac potential in anterior mesoderm. Dev Cell 13: 254–267. 17681136 10.1016/j.devcel.2007.05.012 PMC2709538

[22] Lawson ND, Weinstein BM (2002) In vivo imaging of embryonic vascular development using transgenic zebrafish. Dev Biol 248: 307–318. 12167406 10.1006/dbio.2002.0711

[23] Stern MD (1992) Theory of excitation-contraction coupling in cardiac muscle. Biophys J 63: 497–517. 1330031 10.1016/S0006-3495(92)81615-6 PMC1262173

[24] Arnaout R, Ferrer T, Huisken J, Spitzer K, Stainier DY, et al (2007) Zebrafish model for human long QT syndrome. Proc Natl Acad Sci U S A 104: 11316–11321. 17592134 10.1073/pnas.0702724104 PMC2040896

[25] Bers DM (2002) Cardiac excitation-contraction coupling. Nature 415: 198–205. 11805843 10.1038/415198a

[26] Chi NC, Shaw RM, Jungblut B, Huisken J, Ferrer T, et al (2008) Genetic and physiologic dissection of the vertebrate cardiac conduction system. PLoS Biol 6: e109 doi:10.1371/journal.pbio.0060109 18479184 10.1371/journal.pbio.0060109 PMC2430899

[27] Burns CG, MacRae CA (2006) Purification of hearts from zebrafish embryos. Biotechniques 40 274, 276, 278 passim. 16568816

[28] Harvey RP, Meilhac SM, Buckingham ME (2009) Landmarks and lineages in the developing heart. Circ Res 104: 1235–1237. 19498208 10.1161/CIRCRESAHA.109.199729

[29] Evans SM, Yelon D, Conlon FL, Kirby ML (2010) Myocardial lineage development. Circ Res 107: 1428–1444. 21148449 10.1161/CIRCRESAHA.110.227405 PMC3073310

[30] Chi NC, Shaw RM, De Val S, Kang G, Jan LY, et al (2008) Foxn4 directly regulates tbx2b expression and atrioventricular canal formation. Genes Dev 22: 734–739. 18347092 10.1101/gad.1629408 PMC2275426

[31] Pellegrini L, Burke DF, von Delft F, Mulloy B, Blundell TL (2000) Crystal structure of fibroblast growth factor receptor ectodomain bound to ligand and heparin. Nature 407: 1029–1034. 11069186 10.1038/35039551

[32] Schlessinger J, Plotnikov AN, Ibrahimi OA, Eliseenkova AV, Yeh BK, et al (2000) Crystal structure of a ternary FGF-FGFR-heparin complex reveals a dual role for heparin in FGFR binding and dimerization. Mol Cell 6: 743–750. 11030354 10.1016/s1097-2765(00)00073-3

[33] Yayon A, Klagsbrun M, Esko JD, Leder P, Ornitz DM (1991) Cell surface, heparin-like molecules are required for binding of basic fibroblast growth factor to its high affinity receptor. Cell 64: 841–848. 1847668 10.1016/0092-8674(91)90512-w

[34] Marques SR, Lee Y, Poss KD, Yelon D (2008) Reiterative roles for FGF signaling in the establishment of size and proportion of the zebrafish heart. Dev Biol 321: 397–406. 18639539 10.1016/j.ydbio.2008.06.033 PMC2752040

[35] Reifers F, Walsh EC, Leger S, Stainier DY, Brand M (2000) Induction and differentiation of the zebrafish heart requires fibroblast growth factor 8 (fgf8/acerebellar). Development 127: 225–235. 10603341 10.1242/dev.127.2.225

[36] Kamimura K, Rhodes JM, Ueda R, McNeely M, Shukla D, et al (2004) Regulation of Notch signaling by Drosophila heparan sulfate 3-O sulfotransferase. J Cell Biol 166: 1069–1079. 15452147 10.1083/jcb.200403077 PMC2172002

[37] Holley SA, Geisler R, Nusslein-Volhard C (2000) Control of her1 expression during zebrafish somitogenesis by a delta-dependent oscillator and an independent wave-front activity. Genes Dev 14: 1678–1690. 10887161 PMC316735

[38] Stainier DY, Lee RK, Fishman MC (1993) Cardiovascular development in the zebrafish. I. Myocardial fate map and heart tube formation. Development 119: 31–40. 8275863 10.1242/dev.119.1.31

[39] Walsh EC, Stainier DY (2001) UDP-glucose dehydrogenase required for cardiac valve formation in zebrafish. Science 293: 1670–1673. 11533493 10.1126/science.293.5535.1670

[40] Beis D, Bartman T, Jin SW, Scott IC, D'Amico LA, et al (2005) Genetic and cellular analyses of zebrafish atrioventricular cushion and valve development. Development 132: 4193–4204. 16107477 10.1242/dev.01970

[41] Westin J, Lardelli M (1997) Three novel Notch genes in zebrafish: implications for vertebrate Notch gene evolution and function. Dev Genes Evol 207: 51–63. 20607480 10.1007/s004270050091

[42] Collery RF, Link BA (2011) Dynamic smad-mediated BMP signaling revealed through transgenic zebrafish. Dev Dyn 240: 712–722. 21337469 10.1002/dvdy.22567 PMC3072245

[43] Sehnert AJ, Huq A, Weinstein BM, Walker C, Fishman M, et al (2002) Cardiac troponin T is essential in sarcomere assembly and cardiac contractility. Nat Genet 31: 106–110. 11967535 10.1038/ng875

[44] Chocron S, Verhoeven MC, Rentzsch F, Hammerschmidt M, Bakkers J (2007) Zebrafish Bmp4 regulates left-right asymmetry at two distinct developmental time points. Dev Biol 305: 577–588. 17395172 10.1016/j.ydbio.2007.03.001

[45] Stickney HL, Imai Y, Draper B, Moens C, Talbot WS (2007) Zebrafish bmp4 functions during late gastrulation to specify ventroposterior cell fates. Dev Biol 310: 71–84. 17727832 10.1016/j.ydbio.2007.07.027 PMC2683675

[46] Jones CM, Lyons KM, Lapan PM, Wright CV, Hogan BL (1992) DVR-4 (bone morphogenetic protein-4) as a posterior-ventralizing factor in Xenopus mesoderm induction. Development 115: 639–647. 1425343 10.1242/dev.115.2.639

[47] Hild M, Dick A, Rauch GJ, Meier A, Bouwmeester T, et al (1999) The smad5 mutation somitabun blocks Bmp2b signaling during early dorsoventral patterning of the zebrafish embryo. Development 126: 2149–2159. 10207140 10.1242/dev.126.10.2149

[48] Nguyen VH, Schmid B, Trout J, Connors SA, Ekker M, et al (1998) Ventral and lateral regions of the zebrafish gastrula, including the neural crest progenitors, are established by a bmp2b/swirl pathway of genes. Dev Biol 199: 93–110. 9676195 10.1006/dbio.1998.8927

[49] Nikaido M, Tada M, Saji T, Ueno N (1997) Conservation of BMP signaling in zebrafish mesoderm patterning. Mech Dev 61: 75–88. 9076679 10.1016/s0925-4773(96)00625-9

[50] Garrity DM, Childs S, Fishman MC (2002) The heartstrings mutation in zebrafish causes heart/fin Tbx5 deficiency syndrome. Development 129: 4635–4645. 12223419 10.1242/dev.129.19.4635

[51] Hurlstone AF, Haramis AP, Wienholds E, Begthel H, Korving J, et al (2003) The Wnt/beta-catenin pathway regulates cardiac valve formation. Nature 425: 633–637. 14534590 10.1038/nature02028

[52] Totong R, Schell T, Lescroart F, Ryckebusch L, Lin YF, et al (2011) The novel transmembrane protein Tmem2 is essential for coordination of myocardial and endocardial morphogenesis. Development 138: 4199–4205. 21896630 10.1242/dev.064261 PMC3171221

[53] Smith KA, Lagendijk AK, Courtney AD, Chen H, Paterson S, et al (2011) Transmembrane protein 2 (Tmem2) is required to regionally restrict atrioventricular canal boundary and endocardial cushion development. Development 138: 4193–4198. 21896629 10.1242/dev.065375

[54] Neugebauer JM, Amack JD, Peterson AG, Bisgrove BW, Yost HJ (2009) FGF signalling during embryo development regulates cilia length in diverse epithelia. Nature 458: 651–654. 19242413 10.1038/nature07753 PMC2688717

[55] Zhao L, Zhao X, Tian T, Lu Q, Skrbo-Larssen N, et al (2008) Heart-specific isoform of tropomyosin4 is essential for heartbeat in zebrafish embryos. Cardiovasc Res 80: 200–208. 18583338 10.1093/cvr/cvn177

[56] Huang W, Zhang R, Xu X (2009) Myofibrillogenesis in the developing zebrafish heart: A functional study of tnnt2. Dev Biol 331: 237–249. 19427304 10.1016/j.ydbio.2009.04.039 PMC2857981

[57] Lackey DP, Carruth ED, Lasher RA, Boenisch J, Sachse FB, et al (2011) Three-dimensional modeling and quantitative analysis of gap junction distributions in cardiac tissue. Ann Biomed Eng 39: 2683–2694. 21822740 10.1007/s10439-011-0369-3

[58] Corti P, Young S, Chen CY, Patrick MJ, Rochon ER, et al (2011) Interaction between alk1 and blood flow in the development of arteriovenous malformations. Development 138: 1573–1582. 21389051 10.1242/dev.060467 PMC3062425

[59] Essner JJ, Branford WW, Zhang J, Yost HJ (2000) Mesendoderm and left-right brain, heart and gut development are differentially regulated by pitx2 isoforms. Development 127: 1081–1093. 10662647 10.1242/dev.127.5.1081

[60] Jou CJ, Spitzer KW, Tristani-Firouzi M (2010) Blebbistatin effectively uncouples the excitation-contraction process in zebrafish embryonic heart. Cell Physiol Biochem 25: 419–424. 20332622 10.1159/000303046 PMC3025892

