# Supplementary figures and images for: 3-OST-7 Regulates BMP-Dependent Cardiac Contraction
Source: PLoS Biol. 2013 Dec 3;11(12):e1001727. doi: 10.1371/journal.pbio.1001727 (PMC3849020; doi:10.1371/journal.pbio.1001727)

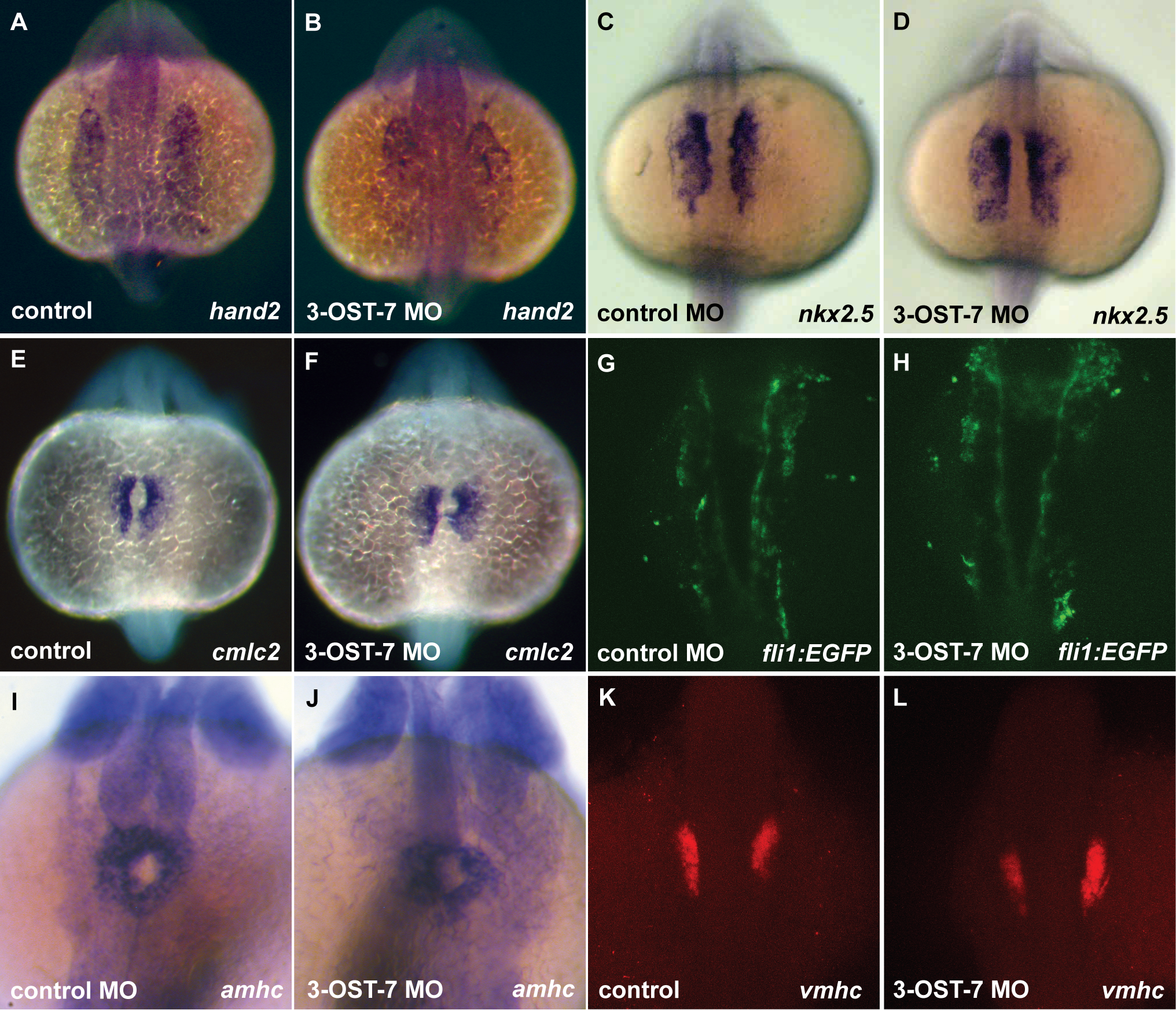

Supplement: Figure S1 — Heart field specification proceeds normally in 3-OST-7 morphants. Dorsal views (anterior on top) of control (uninjected, wild-type) (A, C, E, and K), control MO (injected with control 3-OST-3Z MO) (G and I), and 3-OST-7 morphant (B, D, F, H, J and L) embryos; n = 35 for each group. ISHs for: lateral plate mesoderm marker hand2 (A and B, 17 hpf) and cardiac precursor cell marker nkx2.5 (C and D, 17 hpf), myocardial precursor cell marker cmlc2 (E and F, 17 hpf), atrial precursor cell marker amhc (I and J, 20 hpf), and ventricular precursor cell marker vmhc (K and L, 18 hpf) showed comparable levels and patterns of expression in control and 3-OST-7 morphant embryos. Imaging of fli1 expression in Tg(fli1:EGFP) zebrafish at 18 hpf revealed endocardial lineage is intact in 3-OST-7 morphant embryos (G and H). (TIF) [file pbio.1001727.s001.tif]

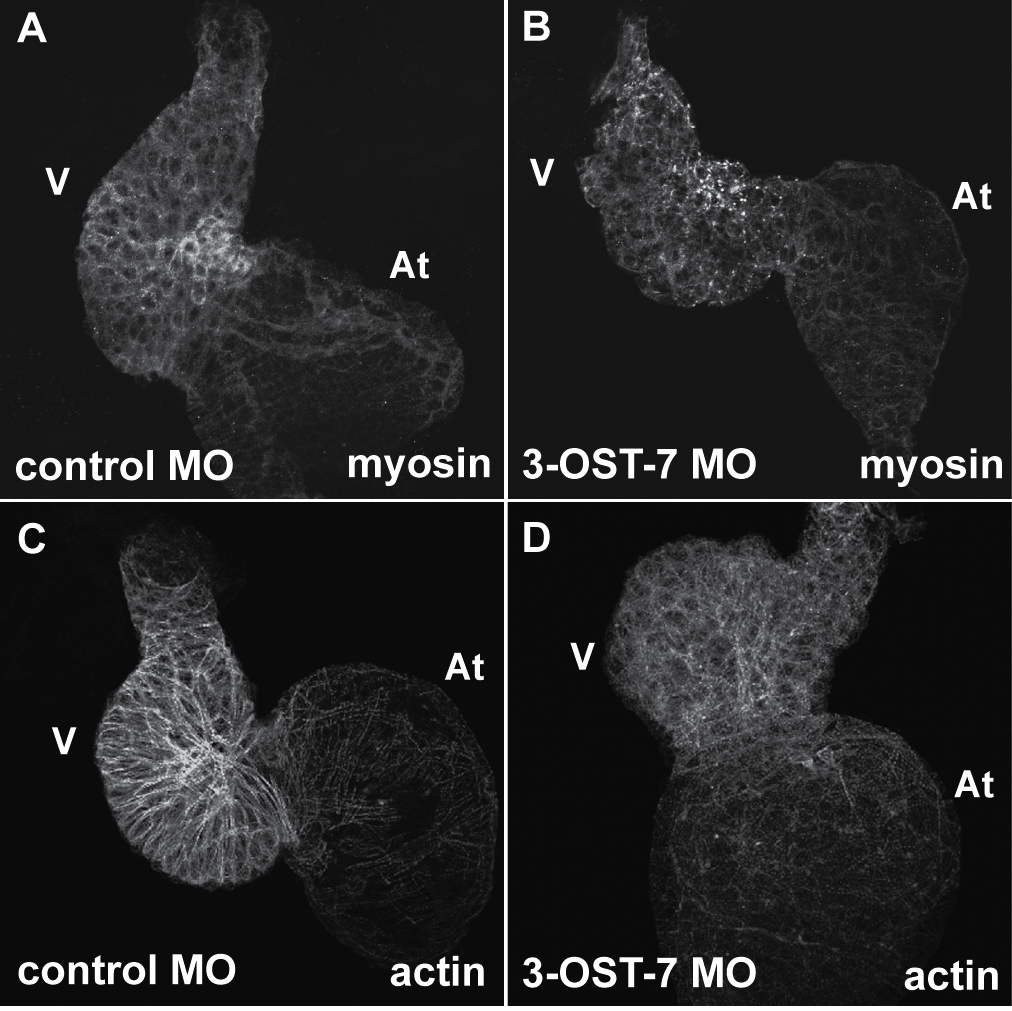

Supplement: Figure S2 — Knockdown of 3-OST-7 disrupts sarcomere organization. Whole mount IHC using anti-myosin (MF20) and phalloidin revealed myosin and actin filaments were disorganized in ventricles of 3-OST-7 morphants (B and D) compared with control (A and C) (n = 30 for each group). At, atrium; V, ventricle. (TIF) [file pbio.1001727.s002.tif]

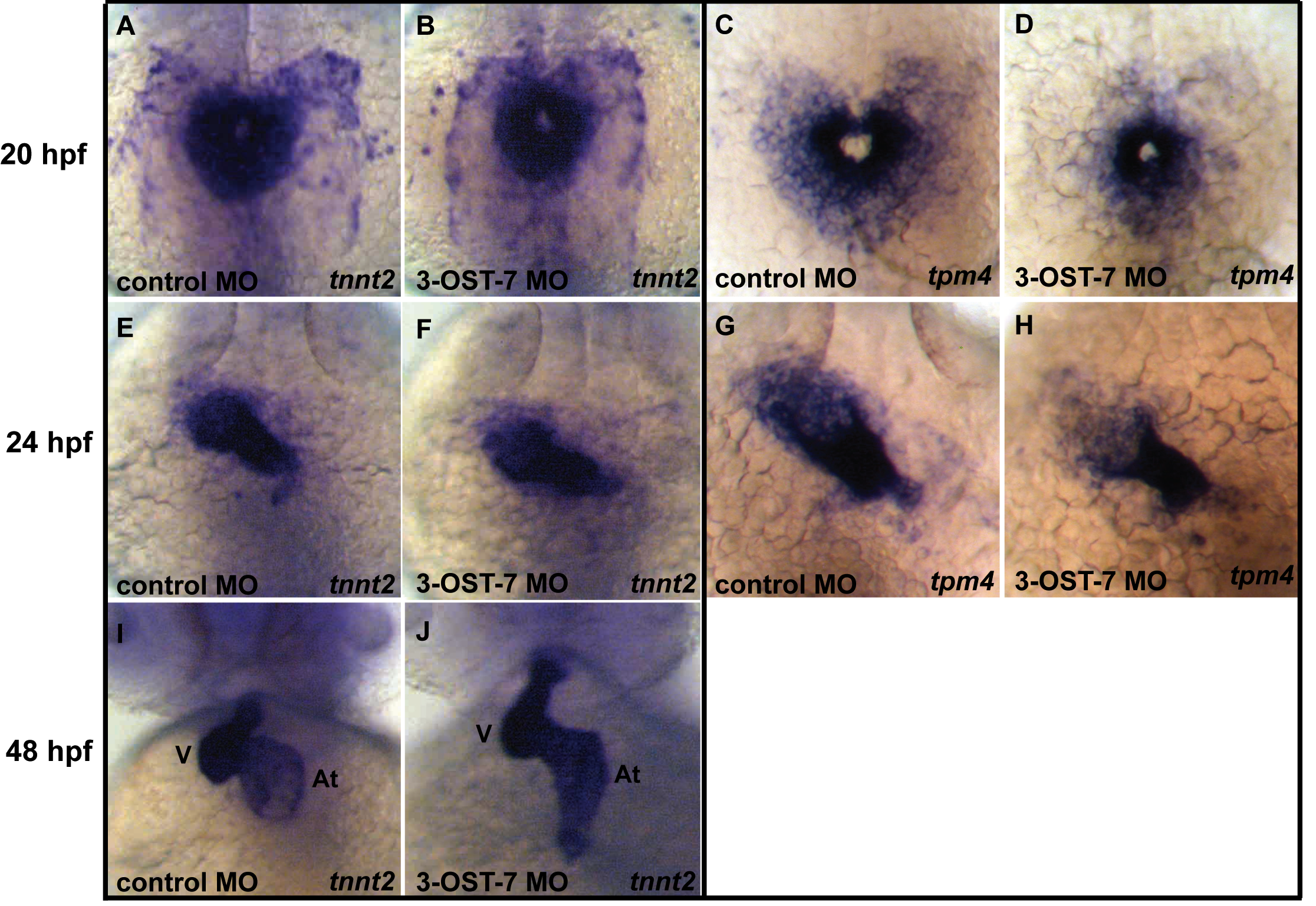

Supplement: Figure S3 — 3-OST-7 controls transcript levels of tpm4 but not those of tnnt2. In situ analysis for tnnt2 showed comparable transcript levels and patterns of expression for 3-OST-7 morphants (B, F, and J) and control (injected with control 3-OST-3Z MO) embryos (A, E, and I) at 20 hpf (A and B), 24 hpf (E and F), and 48 hpf (I and J). In contrast, tpm4 transcripts were decreased in 3-OST-7 morphants (D and H) compared to control embryos (C and G) at 20 hpf (C and D) and 24 hpf (G and H). (A–D) are dorsal views with anterior on top; (E–J) are ventral views with anterior on top; n = 40 for each group. At, atrium; V, ventricle. (TIF) [file pbio.1001727.s003.tif]

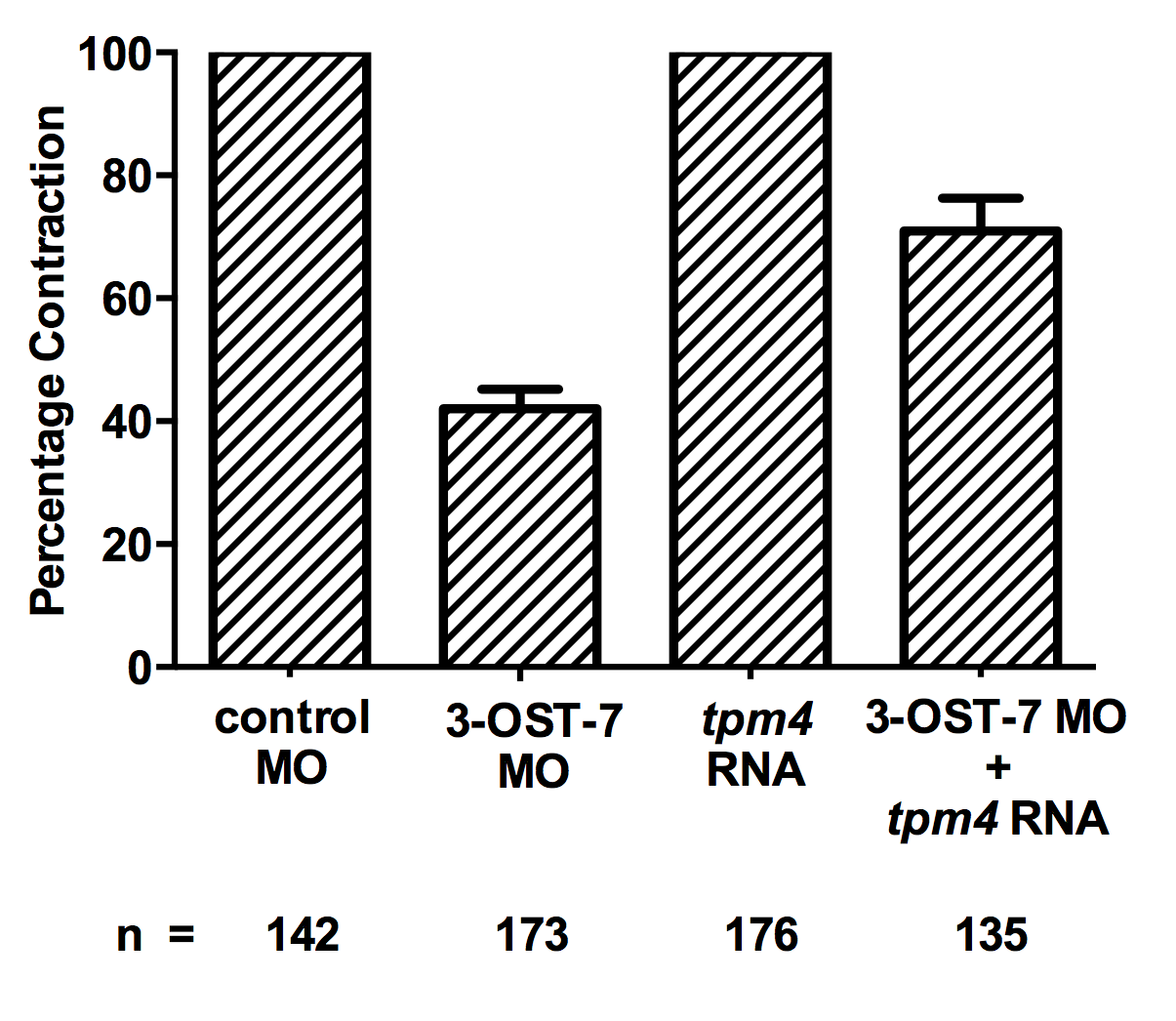

Supplement: Figure S4 — Overexpression of tpm4 rescues the noncontracting ventricle phenotype in 3-OST-7 morphant embryos. Overexpression of tpm4 in control embryos did not alter cardiac function. Strikingly, overexpression in 3-OST-7 morphants rescued ventricular noncontraction (p = 0.0097). (TIFF) [file pbio.1001727.s004.tiff]

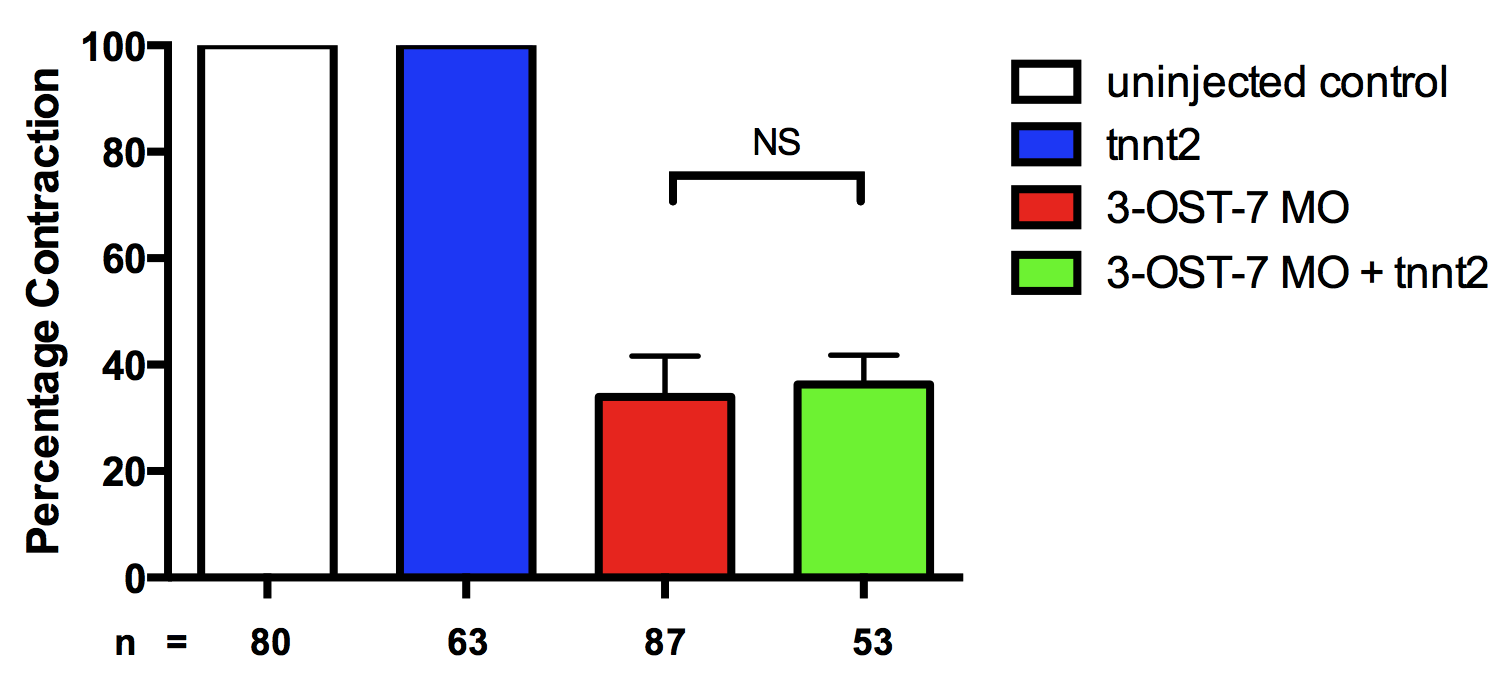

Supplement: Figure S5 — Overexpression of tnnt2 using transient cmlc2:tnnt2-IRES-EGFP plasmid expression does not rescue the noncontracting ventricle phenotype in 3-OST-7 morphant embryos. Tnnt2 transgene expression was scored by EGFP expression. Injection of plasmid alone (blue bar) did not perturb ventricular contraction similar to control (white bar). Injection of both plasmid and 3-OST-7 MO (green bar) resulted in ventricular noncontraction at a percentage similar to injection of 3-OST-7 MO alone (red bar) (NS, p = 0.69). (TIFF) [file pbio.1001727.s005.tiff]

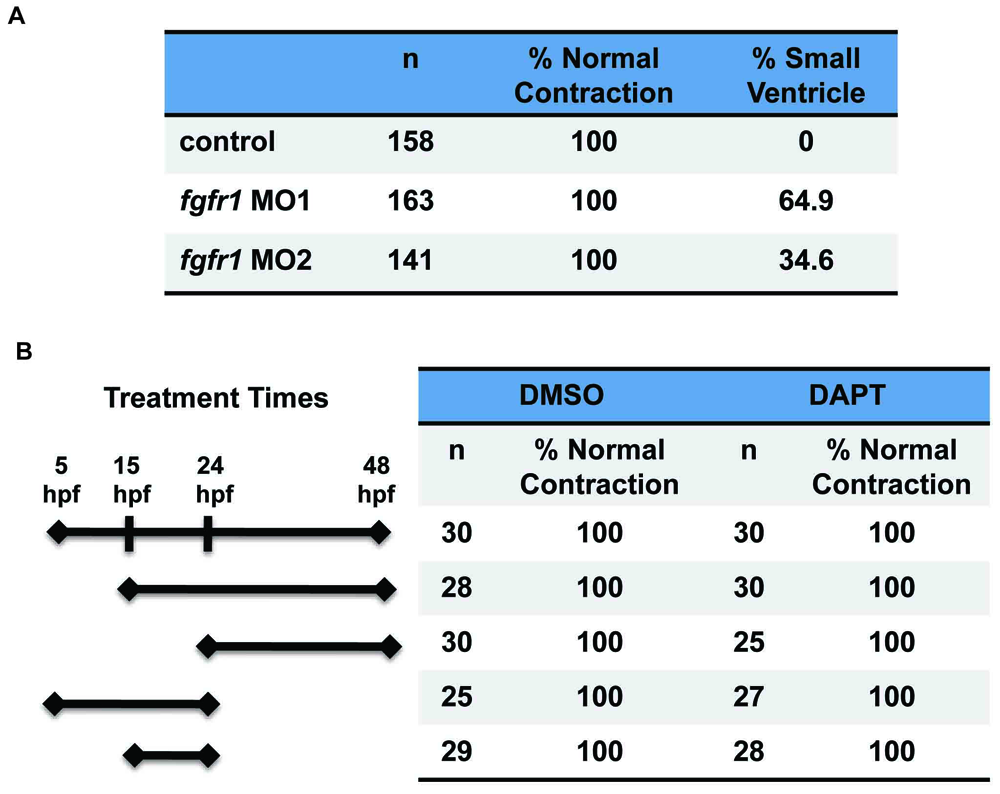

Supplement: Figure S6 — Disrupting the FGF and Notch signaling pathways do not phenocopy the noncontracting ventricle phenotype of 3-OST-7 knockdown. (A) Table showing percentage of normal contraction and small ventricles in control (uninjected) embryos, embryos injected with 4 ng fgfr1 MO1, and embryos injected with 8 ng fgfr1 MO2 at 48 hpf. Ventricular contraction appeared normal in all groups. (B) Timeline showing the time and duration of 75 µm DAPT treatment and table showing percentage of embryos with normal contraction. Control embryos were treated with 0.3% (v/v) DMSO. Ventricular contraction appeared normal in all treatments. To ensure DAPT was working, embryos treated with DAPT starting at 5 hpf were observed at 18 hpf for somite defects. All embryos that received DAPT treatment starting at this timepoint developed somite defects at 18 hpf. No somite disruption was observed in corresponding DMSO treatments. (TIF) [file pbio.1001727.s006.tif]

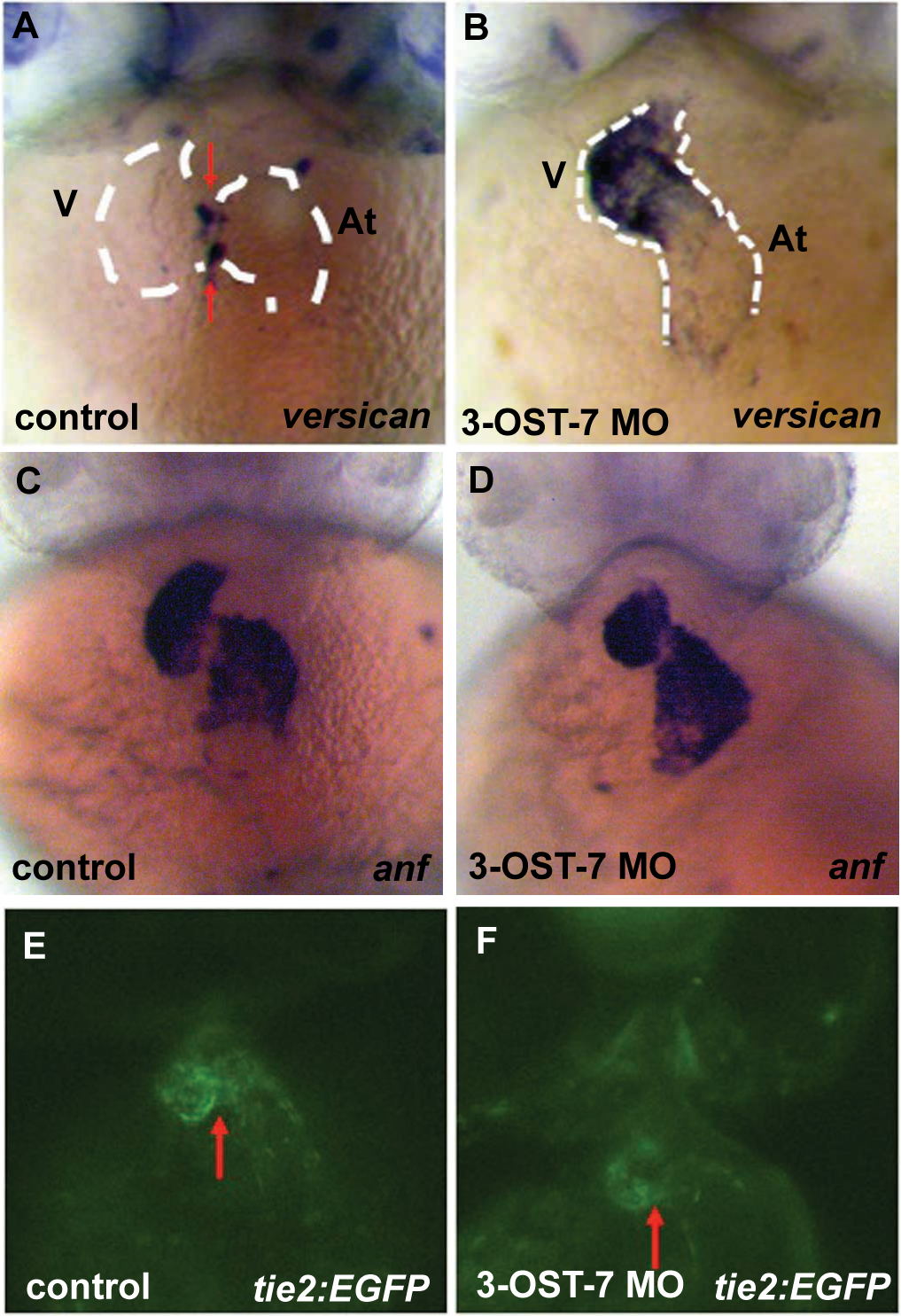

Supplement: Figure S7 — Knockdown of 3-OST-7 affects expression pattern of versican but does not alter expression patterns of other heart differentiation markers at 48 hpf. ISH for versican, an AV myocardium-localized marker, showed ectopic expression in ventricular myocardium of 3-OST-7 morphants (A and B). Expression of anf, a marker for chamber myocardium, was comparable between control and 3-OST-7 morphants (C and D). Tie2 expression, assessed in Tg(tie2:EGFP) embryos, was normally expressed in 3-OST-7 morphant AV endocardium (F) and is similar to control (E). Control groups (A and C) were injected with control 3-OST-3Z MO, control (E) was uninjected. V, ventricle; At, atrium; red arrows point to AV; dashed white lines outline the heart. (TIF) [file pbio.1001727.s007.tif]

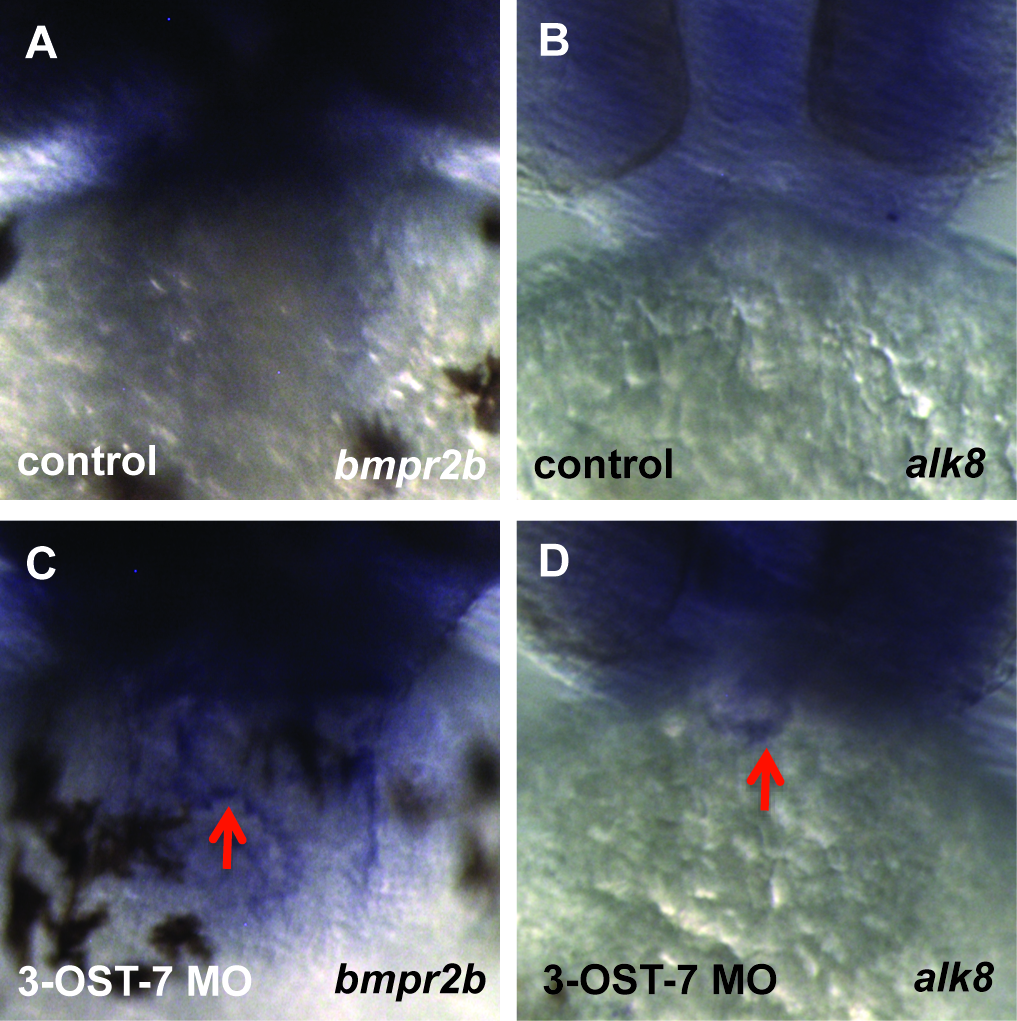

Supplement: Figure S8 — Knockdown of 3-OST-7 alters expression patterns of BMP receptors bmpr2b and alk8 . ISH for bmpr2b (A, C) showed ectopic expression in heart (C, red arrow) of 3-OST-7 morphant. ISH for alk8 (B, D) showed ectopic expression in outflow tract (D, red arrow) of 3-OST-7 morphant. (TIF) [file pbio.1001727.s008.tif]
