# Supplementary material for: 3-OST-7 Regulates BMP-Dependent Cardiac Contraction
Source: PLoS Biol. 2013 Dec 3;11(12):e1001727. doi: 10.1371/journal.pbio.1001727 (PMC3849020; doi:10.1371/journal.pbio.1001727)
Supplement: Table S1 — Action potential parameters recorded from control (uninjected, wild-type) embryos and 3-OST-7 morphants. (DOCX) [file pbio.1001727.s009.docx]

**Table S1. Action potential parameters recorded from control (uninjected, wild-type) embryos and 3-OST-7 morphants.**

| **CONTROL ATRIUM** | | | | | | **3-OST-7 MO ATRIUM** | | | | | |
| --- | --- | --- | --- | --- | --- | --- | --- | --- | --- | --- | --- |
|  | **MDP (mV)** | **APA (mV)** | **CL (ms)** | **APD_50_ (ms)** | **APD_90_ (ms)** |  | **MDP (mV)** | **APA (mV)** | **CL (ms)** | **APD_50_ (ms)** | **APD_90_ (ms)** |
| 1 | -68.48 | 104.90 | 673.90 | 147.70 | 175.70 | 1 | -78.71 | 107.01 | 485.95 | 130.10 | 153.45 |
| 2 | -71.41 | 110.05 | 437.90 | 129.20 | 148.70 | 2 | -57.02 | 66.50 | 408.55 | 131.05 | 169.50 |
| 3 | -72.81 | 102.81 | 472.10 | 131.20 | 153.40 | 3 | -79.70 | 113.34 | 538.00 | 162.60 | 184.05 |
| 4 | -64.29 | 96.66 | 538.50 | 139.20 | 168.10 | 4 | -80.05 | 115.78 | 762.95 | 134.40 | 153.45 |
| 5 | -75.14 | 111.13 | 554.40 | 141.20 | 165.50 |  |  |  |  |  |  |
| **CONTROL VENTRICLE** | | | | | | **3-OST-7 MO VENTRICLE** | | | | | |
|  | **MDP (mV)** | **APA (mV)** | **CL (ms)** | **APD_50_ (ms)** | **APD_90_ (ms)** |  | **MDP (mV)** | **APA (mV)** | **CL (ms)** | **APD_50_ (ms)** | **APD_90_ (ms)** |
| 1 | -79.91 | 115.23 | 444.00 | 243.00 | 280.00 | 1 | -81.94 | 118.09 | 529.75 | 232.45 | 271.40 |
| 2 | -76.63 | 114.36 | 427.40 | 197.00 | 233.00 | 2 | -72.64 | 96.44 | 381.45 | 198.05 | 248.55 |
| 3 | -76.38 | 111.52 | 370.00 | 230.00 | 265.00 | 3 | -77.42 | 111.85 | 474.10 | 250.95 | 284.15 |
| 4 | -75.81 | 116.11 | 540.50 | 267.70 | 305.00 | 4 | -81.81 | 123.09 | 2829.15 | 273.00 | 300.55 |
| 5 | -62.86 | 103.48 | 543.20 | 248.00 | 294.60 |  |  |  |  |  |  |

MDP, maximal diastolic potential; APA, action potential amplitude; CL, cycle length, APD, action potential duration.
