# Supplementary material for: 3-OST-7 Regulates BMP-Dependent Cardiac Contraction
Source: PLoS Biol. 2013 Dec 3;11(12):e1001727. doi: 10.1371/journal.pbio.1001727 (PMC3849020; doi:10.1371/journal.pbio.1001727)
Supplement: Table S2 — Statistical t -test comparison of action potential parameters between control (uninjected, wild-type) embryos and 3-OST-7 morphants. (DOCX) [file pbio.1001727.s010.docx]

**Table S2. Statistical *t*-test comparison of action potential parameters between control (uninjected, wild-type) embryos and 3-OST-7 morphants.**

| **ATRIUM** | | | | | | | | | | |
| --- | --- | --- | --- | --- | --- | --- | --- | --- | --- | --- |
|  | **MDP (mV)** | | **APA (MV)** | | **CL (ms)** | | **APD_50_ (ms)** | | **APD_90_ (ms)** | |
|  | **CONTROL** | **3-OST-7 MO** | **CONTROL** | **3-OST-7 MO** | **CONTROL** | **3-OST-7 MO** | **CONTROL** | **3-OST-7 MO** | **CONTROL** | **3-OST-7 MO** |
| Mean | -70.42 | -73.87 | 105.11 | 100.66 | 535.36 | 548.86 | 137.70 | 139.54 | 162.28 | 165.11 |
| SEM | 1.87 | 5.62 | 2.62 | 11.54 | 40.66 | 76.16 | 3.38 | 7.74 | 4.94 | 7.36 |
| n | 5 | 4 | 5 | 4 | 5 | 4 | 5 | 4 | 5 | 4 |
| *P*(T<=t) two-tail | **0.54** | | **0.69** | | **0.86** | | **0.82** | | **0.75** | |
| **VENTRICLE** | | | | | | | | | | |
|  | **MDP (mV)** | | **APA (mV)** | | **CL (ms)** | | **APD_50_ (ms)** | | **APD_90_ (ms)** | |
|  | **CONTROL** | **3-OST-7 MO** | **CONTROL** | **3-OST-7 MO** | **CONTROL** | **3-OST-7 MO** | **CONTROL** | **3-OST-7 MO** | **CONTROL** | **3-OST-7 MO** |
| Mean | -74.32 | -78.45 | 112.14 | 112.37 | 465.02 | 461.77 | 237.14 | 227.15 | 275.52 | 268.03 |
| SEM | 2.95 | 2.20 | 2.30 | 5.78 | 33.69 | 43.25 | 11.72 | 15.50 | 12.59 | 10.41 |
| n | 5 | 4 | 5 | 4 | 5 | 4 | 5 | 4 | 5 | 4 |
| *P*(T<=t) two-tail | **0.32** | | **0.97** | | **0.37** | | **0.94** | | **0.97** | |

MDP, maximal diastolic potential; APA, action potential amplitude; CL, cycle length, APD, action potential duration; SEM, standard error of the mean.
