# Supplementary material for: 3-OST-7 Regulates BMP-Dependent Cardiac Contraction
Source: PLoS Biol. 2013 Dec 3;11(12):e1001727. doi: 10.1371/journal.pbio.1001727 (PMC3849020; doi:10.1371/journal.pbio.1001727)
Supplement: Table S3 — ISH analysis comparing expression of nine BMP receptors in control wild-type and 3-OST-7 morphants. (DOCX) [file pbio.1001727.s011.docx]

**Table S3*. In situ* hybridization analysis comparing expression of nine BMP receptors in control wildtype and 3-OST-7 morphants.**

| **BMP Receptor** | **17 somite stage** | | **24 hpf** | | **36 hpf** | | **48 hpf** | |
| --- | --- | --- | --- | --- | --- | --- | --- | --- |
|  | **Wildtype Expression** | **Expression in 3-OST-7 MO** | **Wildtype Expression** | **Expression in 3-OST-7 MO** | **Wildtype Expression** | **Expression in 3-OST-7 MO** | **Wildtype Expression** | **Expression in 3-OST-7 MO** |
| ***alk3a*** | Ubiquitous  (n=30) | Slightly increased  (n=30) | Ubiquitous  (n=34) | Decreased  (n=30) | Brain; not expressed in heart  (n=26) | Same  (n=28) | Brain; not expressed in heart  (n=28) | Slightly increased (n=27) |
| ***alk3b*** | Ubiquitous  (n=30) | Increased  (n=32) | Ubiquitous  (n=38) | Same  (n=35) | Brain; not expressed in heart  (n=26) | Same  (n=26) | Brain; not expressed in heart  (n=31) | Same  (n=28) |
| ***alk1*** | Not expressed  (n=32) | Same  (n=30) | Ubiquitous,  very faint  (n=30) | Same  (n=29) | Not expressed  (n=26) | Same  (n=23) | Ubiquitous,  very faint,  (n=30) | Same  (n=25) |
| ***bmpr2b*** | Very faint, ubiquitous; not expressed in heart  (n=30) | Slightly increased; not expressed in heart  (n=29) | Very faint, ubiquitous; not expressed in heart  (n=35) | Increased; not expressed in heart  (n=31) | Brain, otic vesicles; not expressed in heart  (n=27) | Same  (n=25) | Brain; not expressed in heart  (n=22) | Increased (n=26); upregulated in heart (20/26) |
| ***acvr2a*** | Notochord; not expressed in heart  (n=28) | Same  (n=31) | Brain, neural tube; not expressed in heart  (n=32) | Same  (n=29) | Brain; not expressed in heart  (n=29) | Same  (n=30) | Brain; not expressed in heart  (n=30) | Same  (n=28) |
| ***acvr2b*** | Ubiquitous  (n=31) | Same  (n=30) | Ubiquitous  (n=30) | Same  (n=30) | Brain; faintly expressed in trunk; not expressed in heart  (n=24) | Decreased (n=29) | Brain, pectoral fin; not expressed in heart  (n=30) | Same  (n=21) |
| ***alk6a*** | Brain, neural tube, somites; not expressed in heart  (n=28) | Same  (n=33) | Brain, tail tip;  not expressed in heart  (n=28) | Decreased  (n=26) | Brain, tail tip;  not expressed in  heart  (n=29) | Increased  (n=31) | Otic vesicle, arches  (n=30) | Increased  (n=25) |
| ***alk6b*** | Not expressed  (n=29) | Same  (n=31) | Not expressed  (n=24) | Same  (n=27) | Brain  (n=25) | Not expressed  (n=32) | Not expressed  (n=30) | Same  (n=26) |
| ***alk8*** | Ubiquitous; not expressed in heart  (n=27) | Same  (n=33) | Eyes, brain; not expressed in heart  (n=29) | Same  (n=32) | Brain  (n=37) | Same  (n=34) | Brain  (n=31) | Brain (n=25); upregulated in outflow tract (16/25) |
