# Supplementary material for: 3-OST-7 Regulates BMP-Dependent Cardiac Contraction
Source: PLoS Biol. 2013 Dec 3;11(12):e1001727. doi: 10.1371/journal.pbio.1001727 (PMC3849020; doi:10.1371/journal.pbio.1001727)
Supplement: Table S4 — Heat-shock of embryos from Tg(hs:bmp2b)/+×wild-type cross at 24 hpf and 36 hpf. (DOCX) [file pbio.1001727.s012.docx]

**Table S4. Heat-shock of embryos from Tg(hs:bmp2b)/+ x wild-type cross at 24 hpf and 36 hpf.**

|  | without transgene | | with transgene | |
| --- | --- | --- | --- | --- |
|  | n | Percentage Contraction | n | Percentage Contraction |
| 24 hpf | 33 | 100 | 38 | 100 |
| 36 hpf | 35 | 100 | 34 | 100 |
